# Mitochondrial Complex I Is a Global Regulator of Secondary Metabolism, Virulence and Azole Sensitivity in Fungi

**DOI:** 10.1371/journal.pone.0158724

**Published:** 2016-07-20

**Authors:** Mike Bromley, Anna Johns, Emma Davies, Marcin Fraczek, Jane Mabey Gilsenan, Natalya Kurbatova, Maria Keays, Misha Kapushesky, Marta Gut, Ivo Gut, David W. Denning, Paul Bowyer

**Affiliations:** 1 Manchester Fungal Infection Group, Institute of Inflammation and Repair, Faculty of Medicine and Human Sciences, University of Manchester, 2.24 Core technology Building, Grafton St., Manchester, M13 9NT, United Kingdom; 2 National Aspergillosis Centre, University Hospital of South Manchester, University of Manchester, School of Translational Medicine, Manchester Academic Health Science Centre, 2nd Floor Education & Research Centre, University of Manchester, Manchester, M23 9LT, United Kingdom; 3 Centro Nacional de Analisis Genomico, Parc Cientific de Barcelona, Baldiri Reixac, 4, PCB - Tower I, 08028 Barcelona, Spain; 4 The EMBL-European Bioinformatics Institute, Wellcome Trust Genome Campus, Hinxton, Cambridge, CB10 1SD, United Kingdom; Universidade de Sao Paulo, BRAZIL

## Abstract

Recent estimates of the global burden of fungal disease suggest that that their incidence has been drastically underestimated and that mortality may rival that of malaria or tuberculosis. Azoles are the principal class of antifungal drug and the only available oral treatment for fungal disease. Recent occurrence and increase in azole resistance is a major concern worldwide. Known azole resistance mechanisms include over—expression of efflux pumps and mutation of the gene encoding the target protein cyp51a, however, for one of the most important fungal pathogens of humans, *Aspergillus fumigatus*, much of the observed azole resistance does not appear to involve such mechanisms. Here we present evidence that azole resistance in *A*. *fumigatus* can arise through mutation of components of mitochondrial complex I. Gene deletions of the 29.9KD subunit of this complex are azole resistant, less virulent and exhibit dysregulation of secondary metabolite gene clusters in a manner analogous to deletion mutants of the secondary metabolism regulator, LaeA. Additionally we observe that a mutation leading to an E180D amino acid change in the 29.9 KD subunit is strongly associated with clinical azole resistant *A*. *fumigatus* isolates. Evidence presented in this paper suggests that complex I may play a role in the hypoxic response and that one possible mechanism for cell death during azole treatment is a dysfunctional hypoxic response that may be restored by dysregulation of complex I. Both deletion of the 29.9 KD subunit of complex I and azole treatment alone profoundly change expression of gene clusters involved in secondary metabolism and immunotoxin production raising potential concerns about long term azole therapy.

## Introduction

Fungi destroy approximately 10% of crops and are a direct threat to human health. Additionally they are a major component of the soil ecosystem, form an important part of our food chain through fermentation or direct consumption and are used to produce antibacterial and other simple chemical compounds on a massive scale [1–4]. Fungi have long been known to cause life threatening infections of immuno—compromised individuals but it has recently become clear that fungi are far more commonly associated with serious diseases in immune competent or allergic individuals with asthma [5–10]. Current estimates suggest that as many as 2–3 million life threatening fungal diseases occur every year [8]. Mortality for invasive fungal disease is ~50% rising to 88% where azole resistant fungi are involved [11–14]. The global spend on medical antifungals is ~$7.5 billion and agricultural azoles ~$40 billion. Estimated crop loss to fungi is $50–100 billion worldwide depending on weather and the epidemic nature of many fungal infections [15, 16].

The most important, efficacious and widely used antifungal drug class is the azoles. Azoles act via inhibition of the ergosterol biosynthesis pathwayspecifically by binding to the heme moiety of lanosterol 14α-demethylase (*CYP51*), and sterol Δ^22^-desaturase, (*CYP61*) [17–19]. Interaction with *CYP51* results in a decreased availability of ergosterol and accumulation of 14-methylsterols and 3-ketosteroids [20–22]. Ergosterol is a major component of the plasma membrane and secretory vesicles [23]. Resistance to azoles is constantly arising with high levels of resistance to agricultural azoles occurring within as little as 3 year’s exposure and in an estimated 8% of fungi infecting patients within the course of their treatment [24–34]. Many azoles appear to be fungistatic rather than fungicidal depending on the species tested [35–38] and it has been observed that nutritional status and carbon or nitrogen source strongly affects azole potency. Additionally some fungi appear to be able to display adaptive tolerance to azoles again by unknown mechanisms [39]. Most current research into azole efficacy is focussed on monitoring occurrence of resistance and detection of mutations in the CYP51 gene that are likely to affect azole binding. However in a recent survey of azole resistant *A*. *fumigatus* in Manchester >50% of resistant isolates had no mutation in any CYP51 gene [40]. Given the paucity of alternative antifungal therapies it is important to understandpossible routes to resistance in order that we can design strategies to counter azole resistance in both patient and field.

Fungal secondary metabolites are important in the disease process. Secondary metabolism in fungi has been shown to be under the control of carbon, nitrogen and phosphate catabolite repression. In recent years a global regulator of secondary metabolism, LaeA has been described in various species of *Aspergillus* [41]. This regulator coordinates light signals with development and the production of various secondary metabolites. The activity of LaeA, however, is not sufficient to explain all of the observed regulation of secondary metabolism [42].

Complex I is the first step in the respiratory chain and is located in the inner mitochondrial membrane [43]. This large protein complex functions as an NADH:quinone oxidoreductase. In mammals and filamentous fungi the complex exists in active and inactive states and is thought to de-activate during shifts to higher temperatures or shift between active and inactive states during anoxia-hypoxia [44–46]. Recently *Neurospora crassa* has been used as a genetic model for complex I function and the 29.9KD subunit has been shown to control the shift between active and inactive states [44]. Gene knockouts in complex I of *N*. *crassa* do not significantly affect the ability of the fungus to grow normally [45, 46]. In plants complex I has several functions but again appears dispensable for growth, although growth morphology is affected [47]. An additional role of complex I in synthesis of mitochondrial cardiolipin via pantothenate has been demonstrated [48, 49]. Control of mitochondrial respiration is critical when oxygen availability is limited. Complex I produces high levels of reactive oxygen species when the respiratory chain remains active at low oxygen levels [50]. Additionally in higher eukaryotes both complex I and complex III are regulated during hypoxia with complex I being the first control point in reducing cell respiration when oxygen levels fall [51].

A recent paper has demonstrated a role for oxygen sensing in both pathogenicity and azole resistance of the pathogen *A*. *fumigatus* [52]. However no regulation of the mitochondrial respiratory chain was observed in these experiments. Azole resistance has been reported in *C*. *albicans* due to loss of mitochondrial DNA or mitochondrial function and carbon source may affect drug resistance, and virulence in this organism [53–58]. Other reports suggest that uncoupling of oxidative phosphorylation in the mitochondria or defects in mitochondrial fusion may also lead to alterations in azole sensitivity [59, 60].

As it had previously been demonstrated that azole fungicides act in part though interference of oxygen sensing in fungi and as we had recently discovered that REMI induced mitochondrial complex I 29.9KD subunit mutants were partially azole resistant [61] we hypothesised that inappropriate regulation of mitochondrial respiration might contribute to azole action in a manner similar to *C*. *albicans*. Here we demonstrate a role in pathogenicity, azole resistance and regulation of secondary metabolism for the 29.9KD subunit of complex I in *A*.*fumigatus* and present evidence suggesting a similar role for complex I in azole resistance in diverse filamentous fungi. Intriguingly our results show that the same mutations in complex I have opposite effects on azole resistance to those reported in Candida [56–58].

## Materials and Methods

### Strains, plasmids and chemicals

*A*.*fumigatus* isolates were allowed to sporulate from the culture collection stock then stored in many small aliquots at -80°C in PBS containing 0.05% Tween– 20 and 7% DMSO for use throughout these experiments. AF210 (NCPF 7101) and AF210:101 have been previously described [61]. Construction of the KU80 A1160 strain has been previously described [62]. Itraconazole (Janssen Research Foundation, Beerse, Belgium), voriconazole (Pfizer, Sandwich, UK), posaconazole (Schering-Plough Research Institute, Bloomfield, N.J., USA) were dissolved in DMSO and stored in aliquots at -20°C.*N*. *crassa* knock out strains were obtained from the fungal Genetics Stock Centre (Kansas, USA) and are listed in S6 Table.

### Fungal molecular techniques

Fungal DNA transformation and gene knockout was performed as previously described [62]. Primers used for PCR and gene knockout by PCR fusion are listed in S4 Table.

### RNA extraction and RNAseq

RNA was extracted from100mg fungal mycelium using the Qiagen RNAready kit according to manufacturer’s instructions. Three biological replicate samples were used per condition. RNAseq was performed on an Illumina GAII instrument using each of 8 lanes per replicate to give a final read count of 10–20 million 100 bp reads per sample. FASTQs were subjected to quality control (FASTX Toolkit, FASTQ Quality filter with Phred 77 scores of >20) then assembled onto the A1163 genome sequence with Bowtie2 and further processed with Samtools. Replicate datasets were analysed using EDGER or DESEQ to provide statistical support for the fold change values. Data was filtered to remove results with adjusted p—values worse than 0.01 and to remove results where fold change was less that log_2_>2.

### Determination of sub-inhibitory concentration and IC_50_

Fungi were inoculated into the centre of Vogel’s minimal agar plates containing 1% glucose, 50 mM acetate or 100 mM glycerol or Sabouraud dextrose (SAB) agar plates in 9 cm plates. For determination of *N*. *crassa* growth rates 30 cm race tubes were prepared as described (RACE). Various concentrations of inhibitor or azole were incorporated in to the agar at 50°C before it was poured. Growth was measured daily on triplicate plates or tubes with 4 measurements per plate. Growth rate was then plotted against inhibitor concentration and 50% maximal (untreated) growth rate was calculated. The asymptote on the x—axis represented IC_50_. A similar process was followed to determine sub—inhibitory concentration. In this case maximal growth rate was calculated and the maximum concentration of inhibitor allowing 95% maximal growth rate was determined.

### Determination of mutant virulence in a mouse model

The experiment was performed under UK Home Office project license PPL40/3101 and approved by The University of Manchester Ethics Committee. Male CD1 mice weighing from 20 to 25g (Charles River Ltd) were stored as groups of 5 in vented HEPA-filtered cages with free access to food and water ad libitum. All mice were given enrofloxacin (50 ppm) in their drinking water throughout the course of the study. Mice were immunosuppressed byintraperitoneal injections of 250mg/kg cyclophosphamide and subcutaneous injections of 250 mg/kg cortisone acetate on days −2 and +3. For these studies, *A*. *fumigatus* was cultured on SAB agar at 37°C for 10 days. Conidia were harvested by flooding with phosphate buffered saline containing 0.05% Tween 80. A conidial suspension containing 1×10^9^ conidia/mL was prepared in phosphate buffered saline (Invitrogen, Paisley, UK). Mice were exposed to 12 mL of this solution, which was nebulized (Hudson RCI, High Wycombe, UK) at 1 bar for 1 h. The experiment was continued 14 days post inoculation. The survival post infection of parental, mutant and reconstituted strains was compared using Mann-Whitney U tests. Kaplan-Meier survival analysis was performed and plotted into a graph. Analyses were completed using Stats Direct (Altrincham, Manchester UK).

### Preparation of mitochondria and sub-mitochondrial particles

Mitochondria were used for preparation of sub—mitochondrial particles as described [44] with the following modifications. Mitochondria were prepared from actively growing mycelium by snap freezing in liquid N_2_ and grinding to fine powder in a mortar and pestle. Powder was resuspended in 50 mM Tris pH8.0, 0.25M sucrose and 0.1 mM EDTA and cell fragments were removed by centrifugation at 1000 g for 10 min. The resulting supernatant was spun at 30000 xg for 15 min to pellet the crude mitochondrial fraction. Intact mitochondria were then isolated using the Qiagen Qproteome mitochondrial isolation kit (Qiagen, Jena, Germany). Mitochondria were diluted to a protein concentration of 20 mg/ml and the pH was adjusted to 8.5. Sub—mitochondrial particles (SMP) were then prepared by sonication of the mitochondria on ice using 6 30 second pulses with 30 second cooling periods. Resulting SMPs were pelleted by centrifugation at 30,000g for 30 minutes before storage at -80°C. For induction of hypoxic conditions prior to isolation 200 ml SAB broth was placed in a 500 ml Duran bottle with a magnetic stirrer. The culture was grown overnight on a hotplate—stirrer set to 200 rpm and adjusted empirically to maintain a steady temperature of 37°C. The resulting fine mycelia suspension was then used to inoculate further 200 ml cultures maintained in the same manner. After 16 h growth stirring was stopped and 50 ml sterile mineral oil was added as an overlay to the appropriate flasks. Stirring at 150 rpm was gradually restarted to avoid any mixing of the phases. Triplicate aliquots of 20 ml mycelium was removed by stopping stirring and careful pipetting before immediate harvest through Miracloth and freezing in liquid nitrogen. Mitochondrial fragments were then prepared as previously described.

### Enzyme assays

dNADH:Q1 reductase, and HAR reductase activity was measured in the presence and absence of substrates and inhibitors as described by Ushakova et al [44]. dNADH: Protein levels were determined using the Bradford assay [63]. Q1 reductase activity wasmeasured in the presence of 2 mM MgCl_2_, 20 μg/ml alamethicin, 100 μM dNADH and 80 μM Q1. Rotenone sensitivity was measured in the presence of 50 μM rotenone. KMdNADH was calculated from the 1/v: 1/[dNADH] linear relationship in the presence of 80 μM Q1. KMQ1 was calculated 1/v: 1/[Q1] linear relationship in the presence of 120 μM dNADH. I50 Triton X-100 was calculated from 1/v: 1/[Triton X-100] relationship after treatment with 120 μM dNADH and 80 μM Q1. dNADH:HAR reductase activity was measured in the presence of 2 mM MgCl2 and 20 μg/ml alamethicin. For study of active—inactive transitions SMP (50 μg/ml) from parental A1163 Ku80 or reconstituted A1163 Ku80 Δ29.9KD::29.9KD (not shown) and the A1163 Ku80 Δ29.9KD were performed as described by Ushakova et al. [44]. Except that the inactivation temperature used was 42°C. The initial rates of the rotenone-sensitive dNADH:Q1 reductase activity were determined in the presence of 2 mM KCN, 2 mM MgCl_2_, 100 μM dNADH and 60 μM Q1. The 100% activities were 0.364 and 0.070 μmol dNADH/minute per mg protein at 30°C for parental A1163 Ku80 and the A1163 Ku80 Δ29.9KD, respectively. Results for A1163 Ku80 Δ29.9KD::29.9KD were not significantly different to the parental isolate.

### Measurement of ergosterol

Levels of ergosterol were measured essentially as described [64] with the exception that fungi were grown in 10 ml SAB broth with shaking at 37°C for 24 h before harvesting in Miracloth. Triplicate cultures were adjusted to 0, 0.1, 1.0, 4.0 and 8 mg/L itraconazole 4 h prior to harvest then assayed as described.

### Measurement of dissolved oxygen

Dissolved oxygen was measured directly in cultures using a Jenway 970 Portable Dissolved Oxygen Meter and Electrode (Jencons Scientific Ltd, UK). The oxygen meter was calibrated, according to the manufacturer's instructions, to 21% oxygen in water saturated air and to 0% oxygen using the zero salts supplied with the meter.

## Results

### Complex I NADH oxidoredutase 29.9 KD subunit mediates azole resistance in *A*. *fumigatus*

An azole resistant mutant, AF210:101, was isolated as part of an insertional mutant screen in AF210 [56]. The mutant was moderately resistant to itraconazole with an MIC of 2-4mg/l compared to the wild type MIC of 0.25 mg/l. The mutant also displayed moderate resistance to posaconazole, ravuconazole and voriconazole with MIC values being 4 times higher for the mutant in each case. Determination of radial growth rates yielded an IC_50_ for itraconazole of 0.35 mg/l for AF210 and 5.4 mg/l for AF210:101. Subsequent sequencing of the insertion site showed that the plasmid had integrated into the coding region of the AF210 29.9 KD complex I subunit (identical to AFUA_2G10600 in the sequenced Af293 genome). In order to further confirm the association of the mutant phenotype with the NADH oxidoreductase 29.9 KD subunit we transformed AF210:101 with AFUA_2G10600 hosted on the autonomously replicating plasmid pPTRII. Of 7 transformants that were assessed, all showed wild type sensitivity to itraconazole (S1 Fig) suggesting that the observed azole resistance relates directly to inactivation of the 29.9 KD subunit.

A number of available *N*. *Crassa* complex I mutants were also tested for azole resistance (S2 Fig) indicating that many complex I gene knockouts result in azole resistance in this organism, however it is difficult to speculate on the relationship of these knock-outs to resistance as the precise function of each complex I sub—unit is not clear.

To further confirm these findings the 29.9KD subunit was disrupted in a KU80 laboratorystrain, A1160P+ (A1163 KU80) resulting in strain A1163 KU80 Δ29.9KD. Complete removal of the coding region resulted in a strain that had an itraconazole MIC of >8 mg/L (Table 1). Reconstitution of the knockout strain using a zeocin resistance marker resulted in a strain (A1163 KU80 Δ29.9KD::29.9KD) that displayed parental MIC to itraconazole(0.25 mg/L).

**Table 1:** Azole sensitivities of wild-type and mutant strains to itraconazole (ITR), posaconazole (POS) and voriconazole (VOR).

Strain:	MIC_ITR_		MIC_POS_		MIC_VOR_	
	RPMI_GLUC_	RPMI_AC_	RPMI_GLUC_	RPMI_AC_	RPMI_GLUC_	RPMI_AC_
**AF210**	0.25	1.0	0.125	1	0.5	1
**AF210:101** [56]	1	2	0.5	2	2	4
**A1163 KU80**	0.25	2	0.125	1	0.5	1
**A1163 KU80 Δ29.9KD**	>8	>8	4	4	4	4

### Deletion of the complex I 29.9KD subunit does not significantly affect ergosterol level or induction of CYP51A or B gene expression

**Fig 1:**
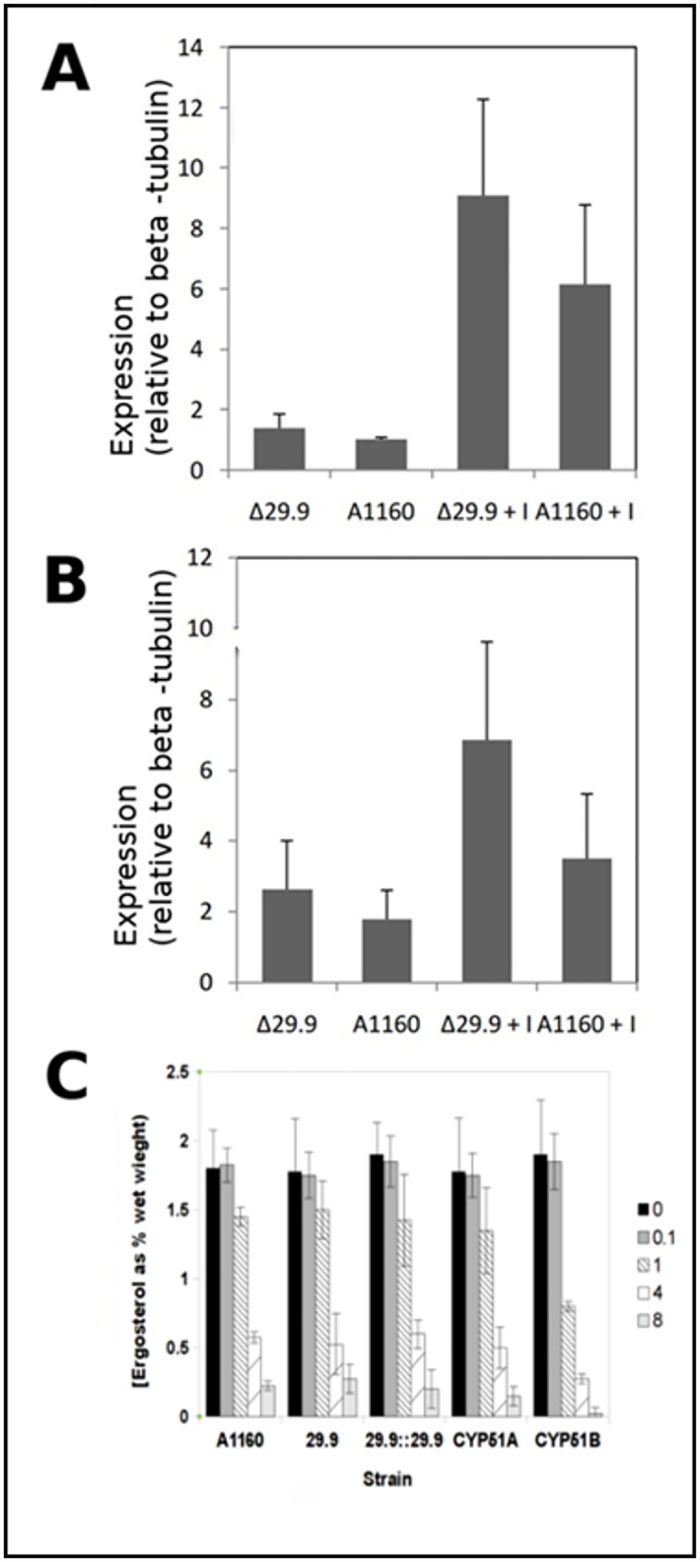
Impact of the 29.9KD deletion on CYP51A and CYP51B expression and ergosterol levels. A. Induction of CYP51A in parental (A1163) and deletion mutant (Δ29.9) isolates 4 h after addition of 1 mg/L itraconazole (Δ29.9+I and A1160+I) or DMSO solvent control (Δ29.9 and A1160). B. Induction of CYP51B in parental (A1160) and deletion mutant (Δ29.9) isolates 4h after addition of 1 mg/L itraconazole (Δ29.9+I and A1160+I) or DMSO solvent control (Δ29.9 and A1160). C. Ergosterol levels of parental (A1160), knockout (Δ29.9) CYP51A (CYP51A) knockout, CYP51B knockout (CYP51B) and reconstituted knockout (Δ29.9::29.9) strains 4 h after addition of a range of itraconazole concentrations (levels given as mg/l in panel C). Results represent averages of three biological replicates with three technical replicates for each. Error bars shown represent standard deviation.

It is known that over—expression of CYP51A or B may lead to azole resistance presumably through an increased level of enzyme being able to support a sufficient supply of ergosterol even in the presence of drug. In order to examine the possibility that complex I knockout or inhibition might affect either CYP51 expression or total cell ergosterol levels we analysed gene expression and ergosterol level of mutant and parental *A*. *fumigatus* during drug challenge. Fig 1A and 1B shows that gene expression for both CYP51A and B is not significantly altered in Δ29.9 mutant strains in comparison with the parental strain. Fig 1C shows that ergosterol levels are not significantly different in the Δ29.9 mutant in comparison to the parental strain at a range of itraconazole concentrations. A control CYP51B knockout strain did show significantly different ergosterol levels at 4 and 8 mg/L itraconazole.

### Chemical inhibition of Complex I NADH oxidoreductase mediates azole resistance in *A*. *fumigatus* and diverse fungi

**Fig 2:**
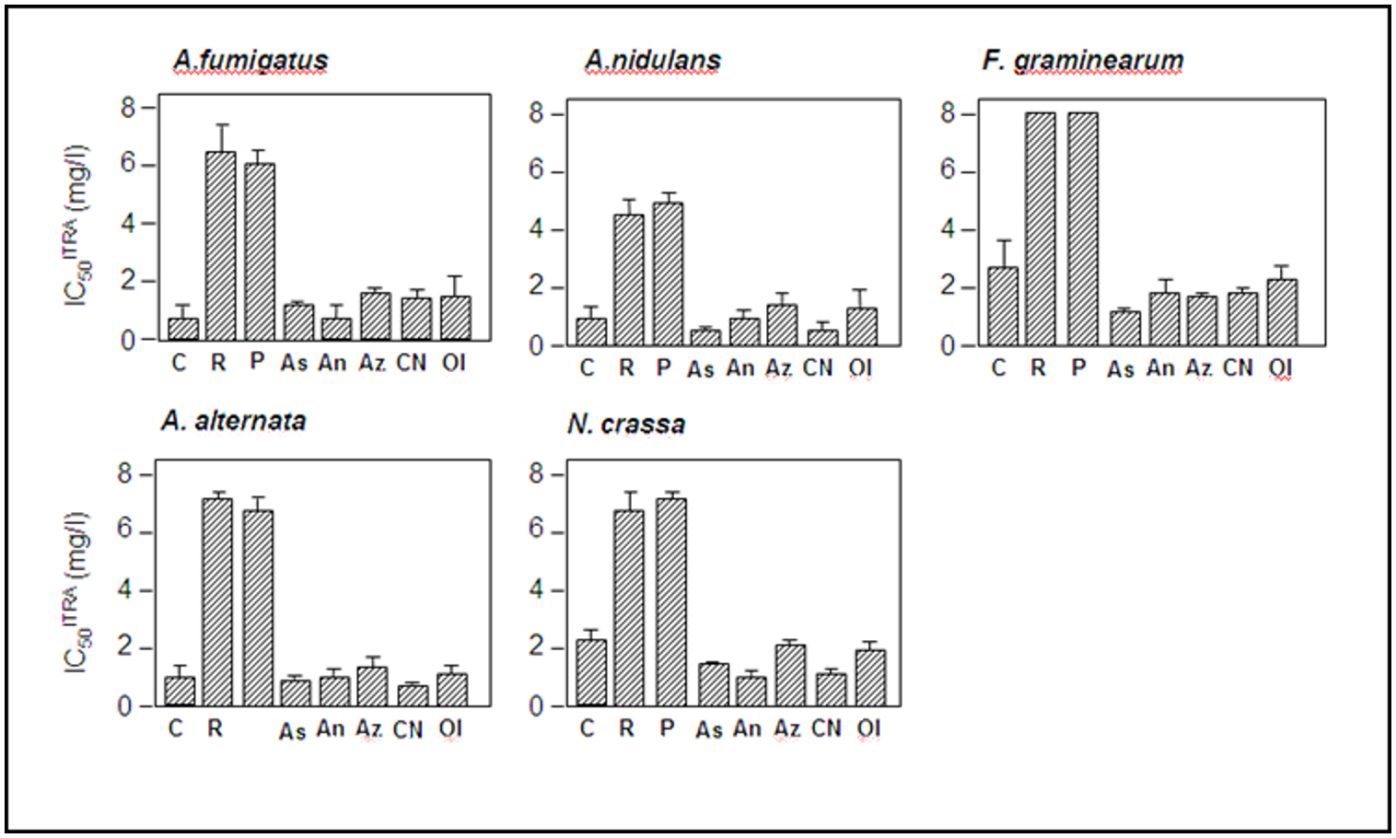
Effect of mitochondrial respiration inhibitors on azole resistance. Itraconazole resistance is shown as IC_50_^ITRA^ of fungi grown on sub-inhibitory concentrations of respiratory inhibitors. Results represent averages of three biological replicates with three technical replicates for each. Error bars represent standard deviation. C: control consisting of addition of water, DMSO or acetone to the medium. Only the water control is shown here for simplicity as all solvent controls showed indistinguishable growth rates. R: Rotenone, P: piericidin A, Az: sodium azide As: azoxystrobin, An: antimycin A, CN: potassium cyanide Ol: oligomycin. Error bars represent standard deviation of IC_50_ values.

A number of inhibitors have been reported to specifically inhibit enzymic function of complex I. Two of these, rotenone and piericidin A were tested to see whether loss or reduction of complex I enzymic function lead to any change in azole resistance. Initially we tested the inhibitors to establish concentrations that did not inhibit growth of *A*. *fumigatus*, *A*. *nidulans*, *Alternaria alternata*, *Fusarium graminearum and Neurospora crassa* (data not shown). In these experiments we defined a sub-inhibitory concentration as one that did not significantly alter radial growth rate by greater than 5% or have any observable effect on gross morphology of hyphae or conidiophores and hyphal inter-branch length. All strains were grown on complete medium in the presence or absence of sub-inhibitory concentrations of either rotenone or piericidin A with the addition of a range of concentrations of itraconazole. As radial growth rate is a universally applicable measure of azole resistance we decided to use this as a comparable measurement for each fungus. IC_50_ values for itraconazole (IC_50_^ITRA^) were then calculated for each fungus in the presence or absence of sub-inhibitory levels of complex I inhibitors (Fig 2). It can be clearly observed that inhibitors of complex I condition azole resistance in each of the fungi tested. For sub-inhibitory concentrations of rotenone the minimum increase in IC_50_^ITRA^ (2.8 fold) was observed in *N*.*crassa* and the maximum (9.1 fold) in *A*. *nidulans*. For sub-inhibitory concentrations of piericidin A the minimum increase in IC_50_^ITRA^ (3.1 fold) was observed in *N*.*crassa* and the maximum (11.3 fold) in *A*. *fumigatus*.

MIC values for itraconazole in the presence of rotenone or piericidin A were also tested for *A*. *fumigatus* AF210 and AF293. Both inhibitors increase the MIC beyond the effective solubility limit of itraconazole (8 mg/l) in this test. This is consistent with our finding thatdeletion of 29.9KD in A1163 KU80 results in resistance to itraconazole (MIC >8 mg/L).

In order to show that azole resistance does not arise from inhibition of respiration generally, we tested inhibitors of complex III (antimycin A, azoxystrobin), complex IV (cyanide, azide) and mitochondrial ATP synthase (oligomycin) in the same manner described above to determine any effects on azole resistance. No significant change in IC_50_^ITRA^ was observed for any of these compounds at any level of growth inhibition (Fig 2). This suggests that the increased azole resistance is specifically mediated by complex I function rather than by an effect on the respiratory chain in general.

### Exposure of *A*. *fumigatus* to rotenone leads to itraconazole resistance in a dose dependent manner

**Fig 3:**
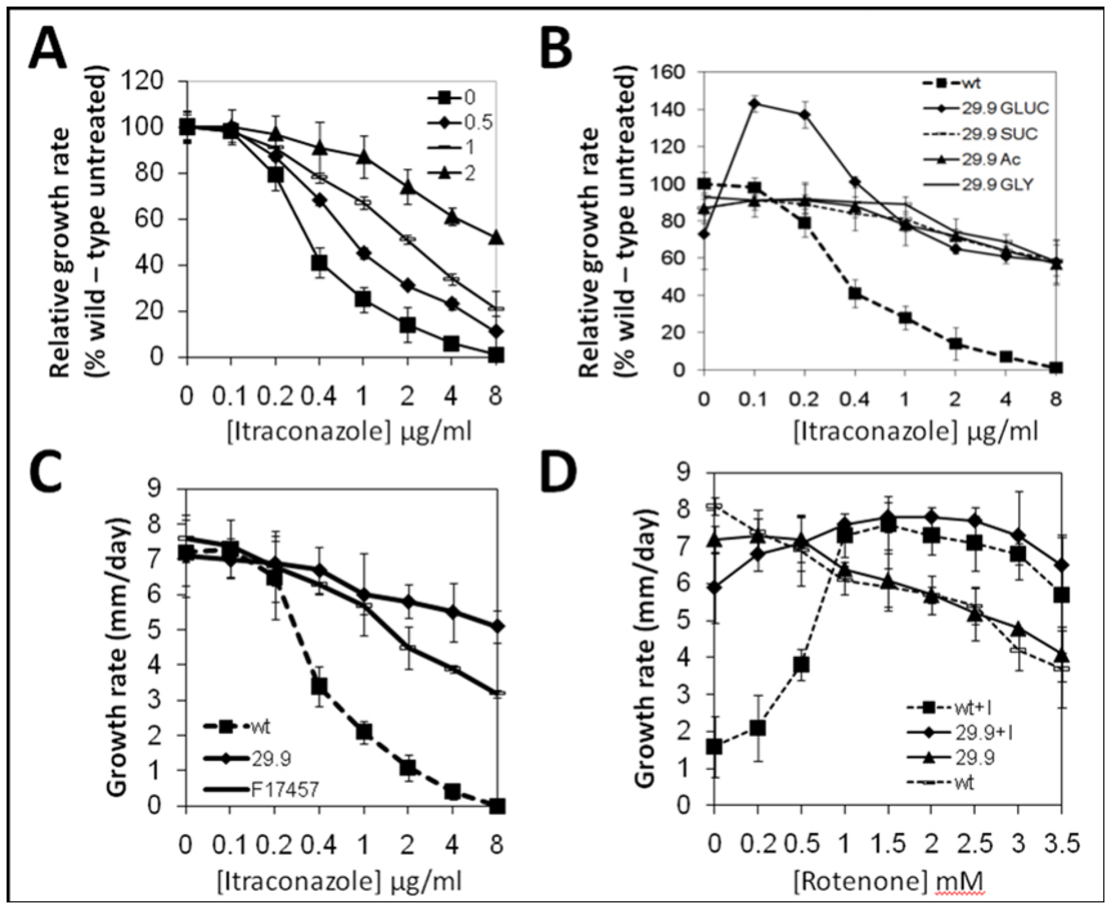
Radial growth rates of parental and 29.9KD NADH dehydrogenase knockout mutants. A. Growth rate of A1163 with increasing levels of itraconazole in the presence of 0, 0.5, 1 and 2 mM rotenone. Growth rates are calculated relative to the no itraconazole control with the appropriate level of rotenone. Results represent averages of four biological replicates with three technical replicates for each. Error bars represent standard deviation. B. Growth rate of parental (wt) and 29.9KD NADH dehydrogenase knockout mutant (29.9) in the presence of increasing levels of itraconazole during growth on 50 mM glucose (GLUC), sucrose (SUC), acetate (Ac) or glycerol (GLY). Growth rates are relative to no ITR on the relevant carbon source. C. Absolute growth rate (mm/day) of the 29.9KD NADH dehydrogenase knockout mutant (29.9), parental control (wt) and a previously described [40] ITR resistant clinical isolate (F17457) in the presence of increasing levels of itraconazole. D. Absolute growth rate (mm/day) of the 29.9KD NADH dehydrogenase knockout mutant (29.9) and parental control (wt) in the presence of increasing levels of rotenone with (+I) or without the addition of 1 mg/L itraconazole.

We tested the effects of increasing rotenone concentrationsand Itraconazole on growth rate of *A*. *fumigatus* on solid agar (Fig 3). These experiments show that increasing rotenone concentration progressively increases resistance of the wild type strain to ITR on complete medium (SAB agar) (Fig 3A). It should be noted that the highest concentration of rotenone tested in this experiment (2 mM) reduced overall growth rate by ~30%.

The addition of low levels of itraconazole to MM + glucose increased the growth rate of the knockout strain beyond that of wild type to a maximum of 140% at 0.1 mg/L itraconazole (Fig 3B). At >1 mg/L itraconazole growth of the knockout on minimal media withglucose matched that on other carbon sources. The 29.9KD knockout showed more rapid growth on high levels of itraconazole than a clinically derived azole resistant isolate (F17457 –now MRMC 17457) that displayed an MIC of >8 mg/L (Fig 3C).

The parental isolate showed growth on 2 mg/L itraconazole indistinguishable from the 29.9KD knockout when 1mM or greater rotenone was included in the SAB agar. Both knockout and parental isolate showed significant but minor increases in growth rate in the presence of > 1 mM rotenone and 2 mg/L itraconazole when compared to growth on rotenone alone (Fig 3D).

### Complex I active/inactive transition is impaired in the 29.9KD knockout

**Fig 4:**
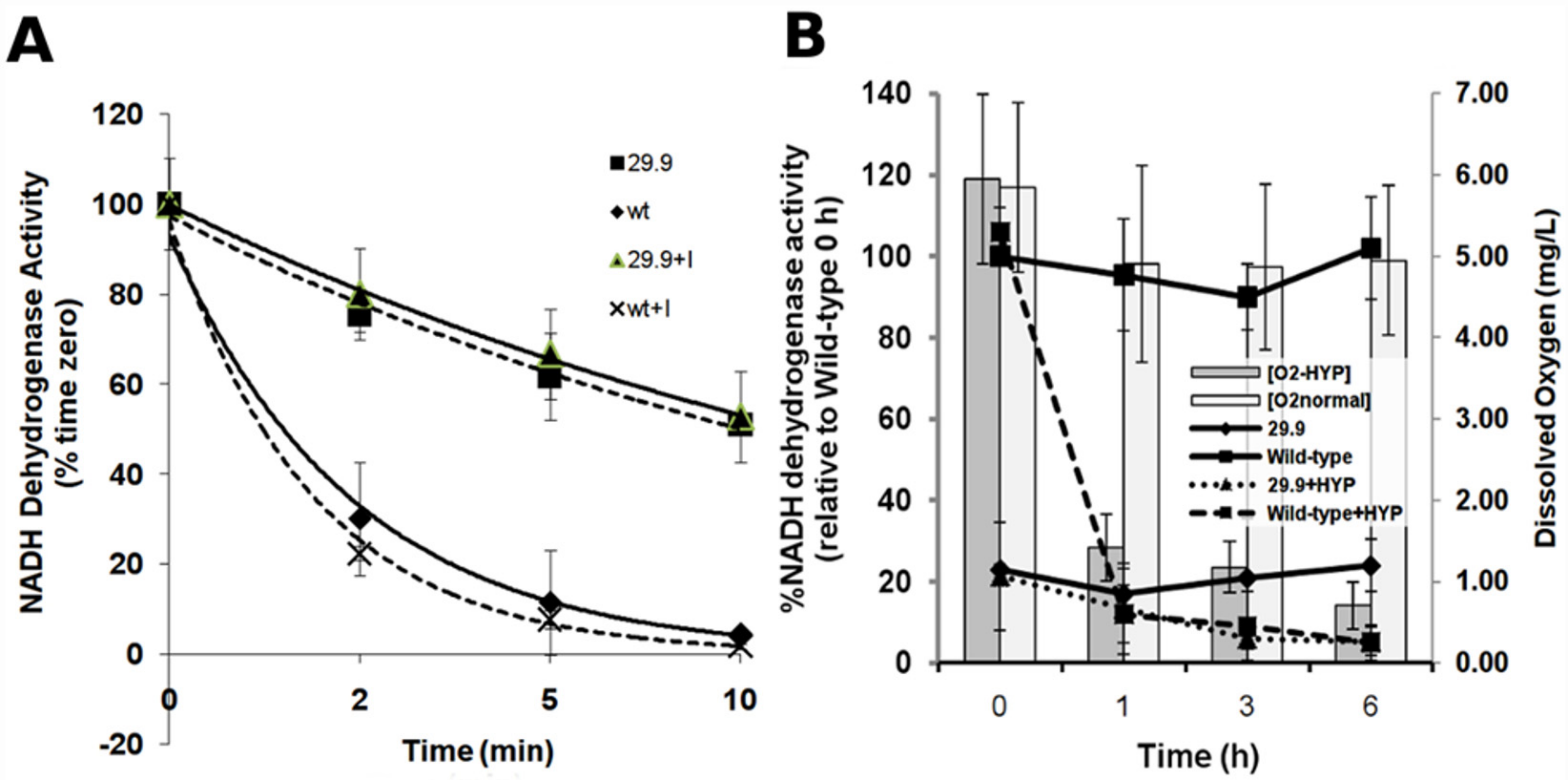
**A. De-activation of complex I NADH dehydrogenase from parental and 29.9KD NADH dehydrogenase knockout strains in the presence or absence of itraconazole.** SMP (50 mg/ml) from parental and the 29.9KD NADH dehydrogenase knockout were pre—incubated with 25 mM dNADH until the substrate was completely oxidised then the incubation was continued at 42°C for 10 minutes. The initial rates of the rotenone-sensitive dNADH:Q1 reductase activity were determined in the presence of 2 mM KCN, 2 mM MgCl_2_, 100 mM dNADH and 50 mM Q1. The 100% activities were 0.315 and 0.12 mmol dNADH/minute per mg protein for the parental and 29.9KD NADH dehydrogenase knockout respectively. For addition of ITR 10μg itraconazole or a control amount of DMSO was added at time zero. B: Activity of complex I NADH dehydrogenase from parental and 29.9KD NADH dehydrogenase knockout strains during normal or hypoxic growth in liquid media. Activities were calculated as described above. Dissolved oxygen levels are shown as bars. O2+HYP: Oxygen level during growth of liquid cultures under mineral oil, O2normal; oxygen level in normal exposed liquid culture, 29.9; NADH dehydrogenase activity for the 29.9KD knockout in normal exposed liquid culture, Wild-type; NADH dehydrogenase activity for the parental strain in normal exposed liquid culture, 29.9+HYP; NADH dehydrogenase activity for the 29.9KD knockout during growth of liquid cultures under mineral oil, Wild-type; NADH dehydrogenase activity for the parental strain during growth of liquid cultures under mineral oil, Results represent the average of three biological experiments with three technical replicates each. Error bars represent standard deviation.

In order to determine that the 29.9 KD complex I subunit of *A*. *fumigatus* functioned in a similar manner to the previously well characterised *N*. *crassa* 29.9 KD subunit we performed NADH dehydrogenase enzyme activity tests on mitochondrial membrane fragments isolated from *A*. *fumigatus* A1163 KU80, A1163 KU80 Δ29.9KD and reconstituted strains. Enzyme activity in the 29.9 KD knockout is 20% of wild—type (Table 2) comparable to the 25% activity seen in the *N*. *Crassa* 29.9 KD knockout. In *N*. *Crassa* this has been suggested to result from lower levels of complex I rather than lower complex I activity [44]. Complex I activity remains relatively high in heat inactivation experiments in the Δ29.9KD strain showing that this subunit is not completely essential for enzyme activity but is rapidly reduced in the parental isolate in a manner comparable to that seen in *N*. *Crassa* and the *N*. *crassa* 29.9KD knockout mutant (Fig 4A) [44–46]. This suggests that the 29.9KD subunit is also responsible for the active-inactive transition in *A*. *fumigatus*. Neither activity nor the thermal inactivation half life are affected when itraconazole is added to the reaction (Fig 4A).

**Table 2:** NADH dehydrogenase enzyme activity in 29.9KD subunit knockout during growth on glucose or acetate.

Enzyme activity (30°C)	A1163 KU80	A1163 KU80 Δ29.9KD		
	Glucose	Acetate	Glucose	Acetate
**dNADH:Q1 reductase (μmol/min/mg total protein)**	0.481 ± 0.071	0.092 ± 0.055	0.024 ± 0.008	0.009 ± 0.027
**dNADH:HAR reductase (μM/min/mg total protein)**	3.221 ± 0.143	1.415± 0.21	0.618 ± 0.045	0.142 ± 0.054
**K**^**dNADH**^ **μM**	7	5	11	12
**K**^**Q1**^ **μM**	32	43	34	39
**I**^**50**^ **Triton X-100 μM**	28	22	41	28
**Rotenone sensitivity (50 μM)**	98%	99%	93%	100 (not detectable)

NADH Q1 dehydrogenase activity is also reduced during growth on a gluconeogenic carbon source such as acetate (Table 2). The activity is highly rotenone sensitive suggesting that the relative insensitivity to rotenone observed for the whole organism may be due to lack of uptake rather than lack of potency although loss of complex I activity does not significantly impair growth.

### Complex I activity is repressed during hypoxic growth

In order to determine whether complex I activity is down regulated during hypoxia we examined activity in parallel cultures in the presence and absence of hypoxic conditions. Spun static cultures retained dissolved oxygen levels close to the starting level whereas those that had been overlayed with 1 cm mineral oil showed a rapid decrease in dissolved oxygen 1 h after overlay (Fig 4B). Overlayed A1160 cultures showed a strong decrease in complex I activity corresponding to reduction in the dissolved oxygen level. The Δ29.9 knockout mutant showed low starting levels of activity that did not decrease as the dissolved oxygen level decreased. Control cultures showed relatively constant levels of both dissolved oxygen and complex I activity throughout the experiment.

### Complex I NADH oxidoreductase 29.9 KD subunit controls expression of secondary metabolism gene clusters in *A*. *fumigatus*

**Fig 5:**
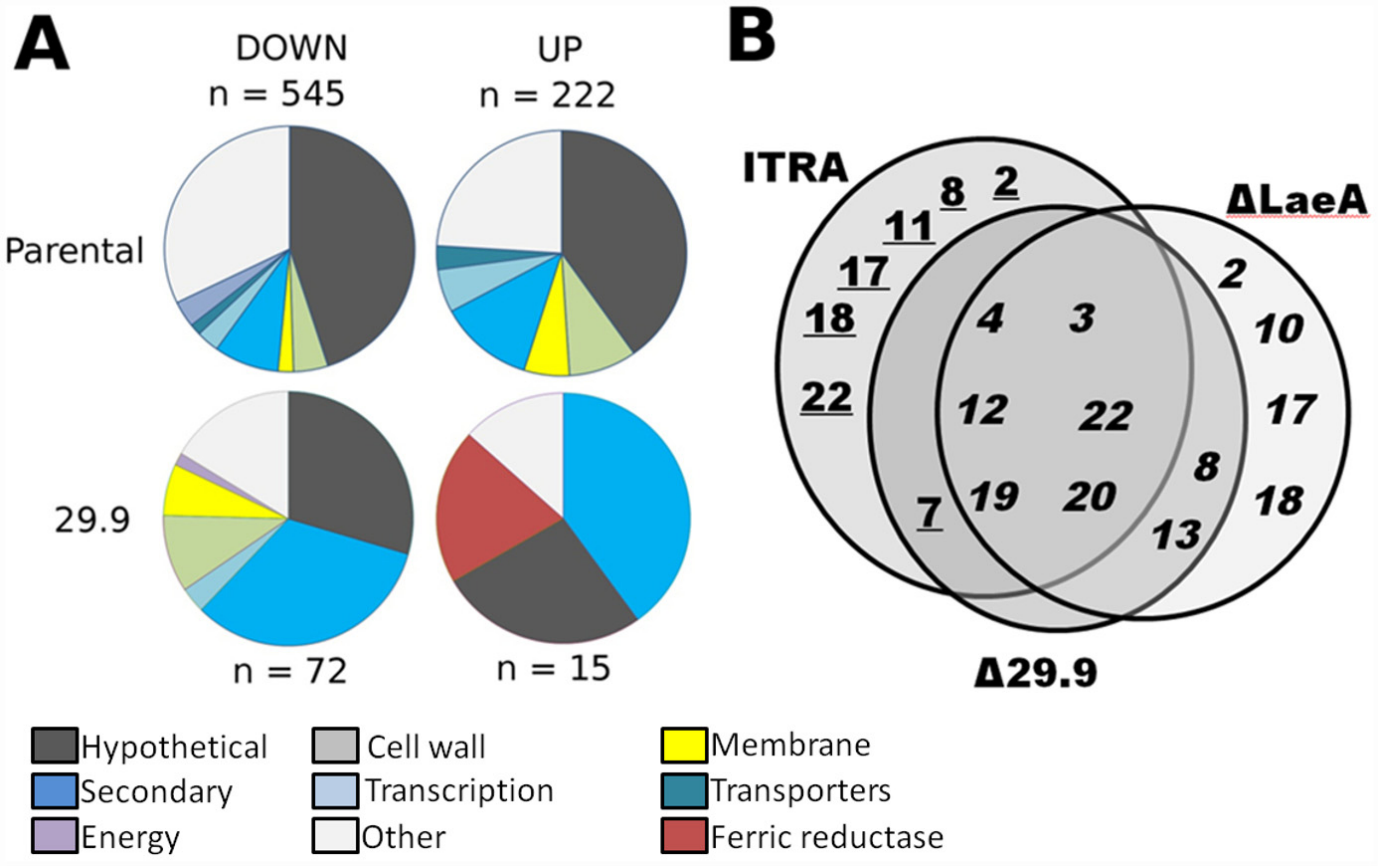
Transcriptional responses of *A*. *fumigatus* to deletion of the complex I 29.9KD subunit and to exposure to Itraconazole. A. Functional classes of genes under and over—regulated by exposure to 1 mg/l itraconazole. This figure shows the total number of genes regulated for each condition. Comparison of expression for the 29.9KD vs paretal transcriptome to identify differences in gene expression due to the 29.9KD knockout are shown in S1–S4 Tables. B. *A*. *fumigatus* gene clusters down—regulated in the 29.9KD NADH oxidoreductase gene and LaeA gene knockouts and during exposure of wild-type *A*. *fumigatus* to itraconazole. Cluster numbers are those given in Khaldi et al. [65]. Numbers indicate those clusters in which ≥50% of genes show >2 fold change in expression in the knockout compared with parental controls. Figures in italics indicate cluster down—regulation whereas numbers of clusters underlined indicate cluster up—regulation.

In order to determine the effect of loss the 29.9KD gene on the *A*. *fumigatus* transcriptome we used RNAseq to quantify gene expression in liquid culture for both parental and the Δ29.9KD strain in the presence and absence of itraconazole (S1–S4 Tables). Genes significantly (p <0.01, FDR <0.01, log_2_ FC >2) over—regulated by deletion of the 29.9KD subunit in the presence and absence of itraconazole in comparison to changes in the parental transcriptome under the same condition were highly enriched for previously defined secondary metabolite gene clusters (Fig 5). For 115 genes significantly down—regulated in the 29.9KD knockout in comparison to the parental transcriptiome in the absence of itraconazole 50 belonged to secondary metabolite clusters (S4 Table). Thirty of 55 genes further down—regulated in the presence of itraconazole belonged to secondary metabolite clusters (S1 Table). Relatively few genes were up—regulated in the 29.9KD knockout when compared to the parental transcriptome. Two of 21 genes up—regulated in the absence of itraconazole (S3 Table) and 7 of 12 genes up—regulated in the presence of itraconazole (S2 Table) compared to the parental transcriptome were recognised secondary metabolite cluster genes (Fig 5). When compared to the only other known global regulator of secondary metabolism, LaeA, we noted that the majority of regulated clusters were also regulated by LaeA (Fig 2) [41]. Further examination of the RNAseq data indicated that LaeA itself shows no change in expression in the 29.9 mutant.

When 29.9KD knockout and parental isolate are challenged with 1 mg/L itraconazole, secondary metabolite clusters designated 3, 10, 15, 19, 22 and 24 contain 38 of the 73 genes that are negatively dysregulated in the mutant and 7 genes of cluster 11 are amongst the 14 genes that are positively dysregulated in the mutant. The effect of itraconazole on expression of secondary metabolite clusters in the parental strain is also dramatic (S3 Fig). Three of the other genes over-expressed in the 29.9KD knockout in comparison to the parental transcriptome are ferric chelate reductases and one is a metal ion transporter (S2 Table).

### Azole resistance and control of secondary metabolism are not mediated by classical catabolite repression or anoxia sensing genes

Our initial hypothesis was that complex I might signal via known carbon catabolite repression or anoxia pathways as reduction in either oxygen or glycolytic carbon source are both expected to reduce complex I activity. In order to test whether the observed 29.9KD knockout phenotype occurred via the classical pathways of catabolite repression or anoxia we constructed gene knockouts for CreA, FacB, AcuM and SREBP. CreA governs carbon catabolite repression in core glycolytic, glyoxylate and gluconeogenic pathways during growth on glycolytic carbon sources such as glucose and FacB together with AcuM regulate activation of the glyoxylate and gluconeogenic pathways during growth on 2-carbon sources such as acetate. SREBP mediates anoxic responses via ergosterol dependent release of a transcriptional activator.

Only the CreA knockout showed increased azole resistance by MIC or radial growth assay (MIC 2 mg/l). The SREBP knockout was highly azole sensitive as previously reported and the MIC_ITR_ was not altered in any of these knockouts by addition of rotenone to the medium.

### The complex I NADH oxidoreductase 29.9KD subunit is required for full virulence in an *A*. *fumigatus* immunocompromised mouse model

**Fig 6:**
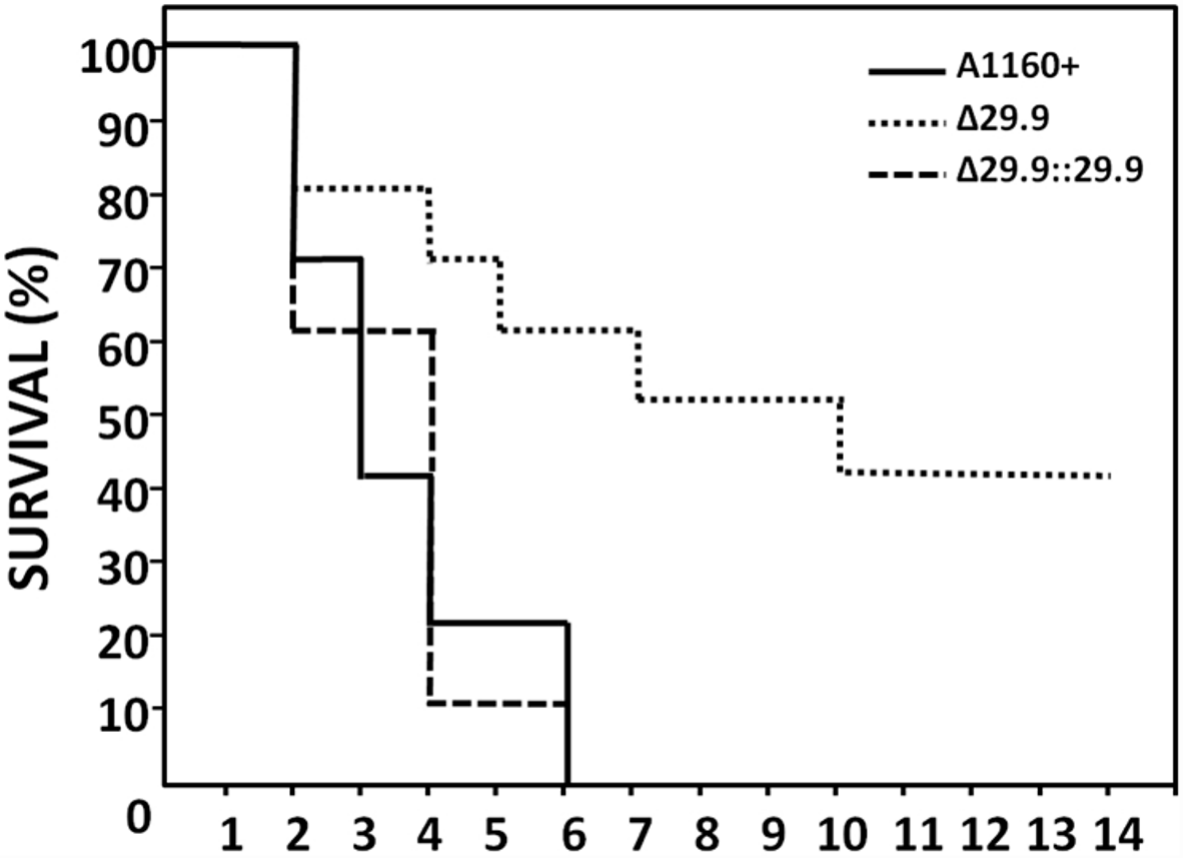
Pathogenicity of mutants, knockouts and reconstituted strains in neutropenic mice. Kaplan Meier plot of survival of mice infected via inhalation.

The association between loss of the 29.9KD complex I subunit and itraconazole resistance may have implications in the pathogenesis of disease in patients undergoing treatment with azoles. To assess the capability of the itraconazole resistant Δ29.9KD to cause disease we assessed its virulence in a neutropenic murine model of invasive aspergillosis (Fig 6). The Δ29.9KD isolate was able to establish an infection that resulted in mortality however it showed attenuated virulence (P = 0.0097; Mann-Whitney U comparison). No difference in virulence was observed between the wild-type and reconstituted (Δ29.9KD::29.9KD) strain (P = 0.927; Mann-Whitney U comparison) (Fig 5). Given the wild-type morphology and growth rate of this mutant we propose that the 29.9KD subunit gene is a virulence determinant in *A*. *fumigatus*.

### The 29.9KD subunit E180D mutation is associated with azole resistance in clinical azole resistant isolates of *A*. *fumigatus*

To determine whether mutations in the 29.9KD complex I subunit are associated with azole resistance in clinical isolates we sequenced the gene in 20 azole sensitive isolates (MIC <0.5 mg/L itraconazole) and 22 resistant isolates (MIC ≥ 8mg/L itraconazole). We observed that a mutation designated by the AF293 contig coordinate 844161 G > T that is predicted to result in an E180D mutation in the 29.9KD subunit protein was present in 12/22 azole resistant isolates and wasobserved in only 2/20 azole sensitive isolates (Table 3). Several isolates used were isolated from the same patient. Assuming that resistance arose separately for these isolates the probability of association between E180D and itraconazole resistance is 0.0028. Assuming that resistance from isolates from a single patient is a single event, the probability of association between E180D and itraconazole resistance is 0.0262.

**Table 3:** Identification of E180D mutations in azole resistant isolates.

Isolate	MIC (itraconazole)	MIC (mg/L) (voriconazole)	MIC (mg/L) (posaconazole)	Mutation in cyp51a	Mutation in 29.9 KD subunit
AF41	0.13	0.5	0.05	nf	nf
F11698	0.25	0.5	0.05	nf	nf
D17	0.5	0.5	0.05	nf	nf
JN10	0.5	0.5	0.25	nf	nf
AF10	0.5	0.5	0.5	nf	nf
AF1163	0.5	0.5	0.05	nf	nf
CEA10	0.5	0.5	0.05	nf	nf
SF2S9	0.5	1	0.25	nf	nf
SF3S1	0.5	1	0.25	nf	nf
SF3S10	0.5	1	0.25	nf	nf
M128	0.5	1	0.25	nf	nf
SF2S6	0.5	1	0.25	nf	nf
SF1S6	0.5	1	0.25	nf	nf
AF210	0.5	1	0.05	nf	nf
AP65	0.5	1	0.25	nf	nf
AF293	0.5	1	0.25	nf	nf
F5211	0.5	1	0.25	nf	E180D
AF300	0.5	1	0.05	nf	nf
SF1S5	0.5	1	0.05	nf	nf
SF4S10	0.5	1	0.05	nf	E180D
F12219	8	0.125	1	G54R	nf
AF72	8	0.5	1	G54E	nf
F14403	8	0.5	8	G54R	nf
F17582	8	1	0.25	nf	E180D
AF90	8	1	1	M220V	nf
F21572	8	1	1	nf	E180D
F7075	8	1	8	G54E	E180D
RSF2S8	8	4	0.05	L98H/TR34	nf
F13619	8	8	0.5	H147Y G448S	nf
F13747	8	8	1	G434C	nf
F15122	8	8	1	G448S	nf
F16311	8	8	1	nf	nf
F12865	8	8	2	G138C	E180D
F13535	8	8	2	G138C	E180D
F16216	8	8	2	L98H/TR34	E180D
F13746	8	8	2	G138C	nf
D1357	8	8	8	L98H/TR34	E180D
F11628	8	8	8	G138C	E180D
F12041	8	8	8	G138C	E180D
F13952	8	8	8	G138C	E180D
F14513	8	8	8	G138C	E180D
F14946	8	8	8	G138C	E180D

Nf, none found.

## Discussion

Here we present evidence that the 29.9KD subunit of complex I is a component in global regulation of secondary metabolite gene clusters, is required for full virulence in a mouse model of invasive aspergillosis, that deletion of this gene results in loss of active—inactive transitions in complex I and that this loss of ability to change state or biochemical inhibition of enzyme activity leads to resistance to azoles. Azole resistance appears to be ergosterol independent and is diametrically opposed to the observation that gene knockout or chemical inhibition of complex I leads to azole sensitivity in Candida.

The range of pathways and phenotypes controlled by the 29.9KD subunit of complex I is similar in scope to that of LaeA [41] with 29.9KD subunit gene deletions deregulating expression of 8 of the 12 LaeA deregulated secondary metabolite clusters during normal growth. One cluster of unknown function (defined as 11 in [65]) is strongly up-regulated in the 29.9KD knockout in the presence of itraconazole. We also noted striking regulation of secondary metabolite clusters during growth in the presence of itraconazole with 6 clusters down-regulated by itraconazole and 6 clusters up-regulated by itraconazole (S3 Fig).

Complex I inactivation through either chemical inhibition by rotenone or piericidin A or deletion of the 29.9 KD complex I subunit leads to azole resistance. We note that insertional inactivation of the gene using REMI resulted in only moderate azole resistance (56). One explanation for this finding is that rotenone or 29.9 KD gene knockout completely inhibits complex I activity biochemically or by preventing reactivation of complex I whereas the AF210:101 29.9 KD mutation allows production of a truncated protein that can partially reactivate complex I. These observations are diametrically opposed to those seen in complex I gene knockouts in *C*. *albicans* [63–68] which lead to dramatically increased azole sensitivity. In *C*. *albicans* complex I knockouts or complete loss of mitochondrial DNA lead to loss of drug efflux gene expression and lower sterol biosynthesis with further effects on iron acquisition systems. In *A*. *fumigatus* complex I mediated azole resistance does not appear to significantly alter ergosterol levels or to increase transcription of key sterol biosynthetic genes.

In addition the intriguing observation that the 29.9 KD knock-out can grow faster in the presence of low azole may result from a compensatory effect of the loss of complex I regulation when ergosterol levels are lowered by drug at sub—MIC levels. Additionally biochemical inhibition of complex I activity results in azole resistance in diverse filamentous ascomycetes, suggesting that this may be a widespread mechanism of azole resistance. Close examination of the observed increase in transcription of efflux transporters in the 29.9KD strain (S3 Table) (AFUA_5G06070, AFUA_6G03080, AFUA_3G07300, AFUA_2G16860) does not support the hypothesis that they are responsiblefor the observed azole resistance in the 29.9KD knockout. AFUA_5G06070, AFUA_6G03080 have been previously knocked out in our laboratory [57] and shown to have no effect on azole resistance. However over-expression might still lead to enhanced drug efflux. When the RNAseq data was examined further we noted that the transporters were expressed at lower levels in the 29.9KD knockout strain in the absence of itraconazole so that their absolute level of expression was lower in the itraconazole- treated 29.9KD mutant than in the itraconazole—treated parental strain. Therefore we suggest that involvement of transporter over-expression as a significant factor in the observed 29.9KD mediated azole resistance remains unproven. Yeasts such as *C*. *albicans* are able to undergo fermentative growth whereas the filamentous ascomycetes are obligate aerobes. Additionally mitochondrial DNA appears to be dispensible in some yeasts but not in filamentous fungi. This implies different regulatory mechanisms for dealing with low oxygen that may explain the diametrically opposed effects of complex I inhibition on azole resistance in *C*. *albicans* where it has been previously shown that 10 of 12 knock out strains in CI increase in azole susceptibility [57]. Khamooshi et al. [59] use transcriptomic profiling to elucidate the pathways and targets of both complex I knockout effects and a controller of complex I phenotypes GOA1. Although some transcriptional pathways such as iron homeostasis are conserved between *A*. *fumigatus* and *C*. *albicans*, other pathways affected appear to be different. We suggest that these differences reflect a potential difference in the role of complex I in these organisms, probably in response to oxygen stress. This would be consistent with overall differences in oxidative metabolism in these organisms such as ability to grow on glycerol or to lose mitochondrial DNA. Other phenotypes such as loss of virulence are conserved in *C*. *albicans* and *A*. *fumigatus*.

Complex I activity is affected by the carbon source on which the fungus is grown with high activity during growth on glucose and very low activity during growth on acetate. Sensitivity to azole is also altered during growth on acetate with 2–4 fold higher MIC. However the MIC does not increase during growth on acetate in the 29.9KD knockout. This suggests that the decreased activity of complex I mediated by the 29.9KD subunit may be responsible for the elevated MIC in these experiments. Carbon starvation has been shown to result in increased transcription of secondary metabolite clusters and the ergosterol pathway in *A*. *nidulans* [66] and anoxia has also been shown to up-regulate ergosterol biosynthesis genes and iron homeostasis pathways [67]. It is not yet clear how this regulation occurs and how nutritional status is sensed. A number of regulators of gluconeogenesis (acuM), the glyoxylate cycle (facB) and ethanol catabolism (alcR) were deleted but found to have no significant effect on the complex I mediated azole resistance phenotype. In contrast deletion of CreA resulting in a defect in catabolite repression slightly elevated the observed MIC_ITR_ suggesting that complex I may exert some of its actions via catabolite control mechanisms.

The loss of virulence observed for the *A*. *fumigatus* 29.9KD mutant should inhibit the development of azole resistance during infection via this mechanism. However the observation of a significant association between the E180D mutation and azole resistance in azole resistant clinical isolates with no target cyp51A mutations suggests that it might support development of higher level resistance in a non-cyp51A dependent manner. The consequences of the E180D mutation on complex I function are unknown. Fungi are known to possess several alternative respiratory pathways including complex II (succinate dehydrogenase), alternative oxidase and possibly other NADH oxidoreductases hence the lack of observable growth effects from complex I inhibition or mutation. Itraconazole does not appear to affect the kinetics of complex I active-inactive transition. Therefore it is unlikely that azole action is mediated via direct inhibition of complex I. Inhibition of complex III, complex IV or the mitochondrial ATP synthase do not alter azole resistance, thus it is unlikely that subtle effects on respiration account for the observed azole resistance. Given that azoles are known to bind to the CYP51A protein and that ergosterol biosynthesis is unaffected in complex I mutants it is unlikely that complex I mediates azole resistance by alteration in ergosterol biosynthesis.

**Fig 7:**
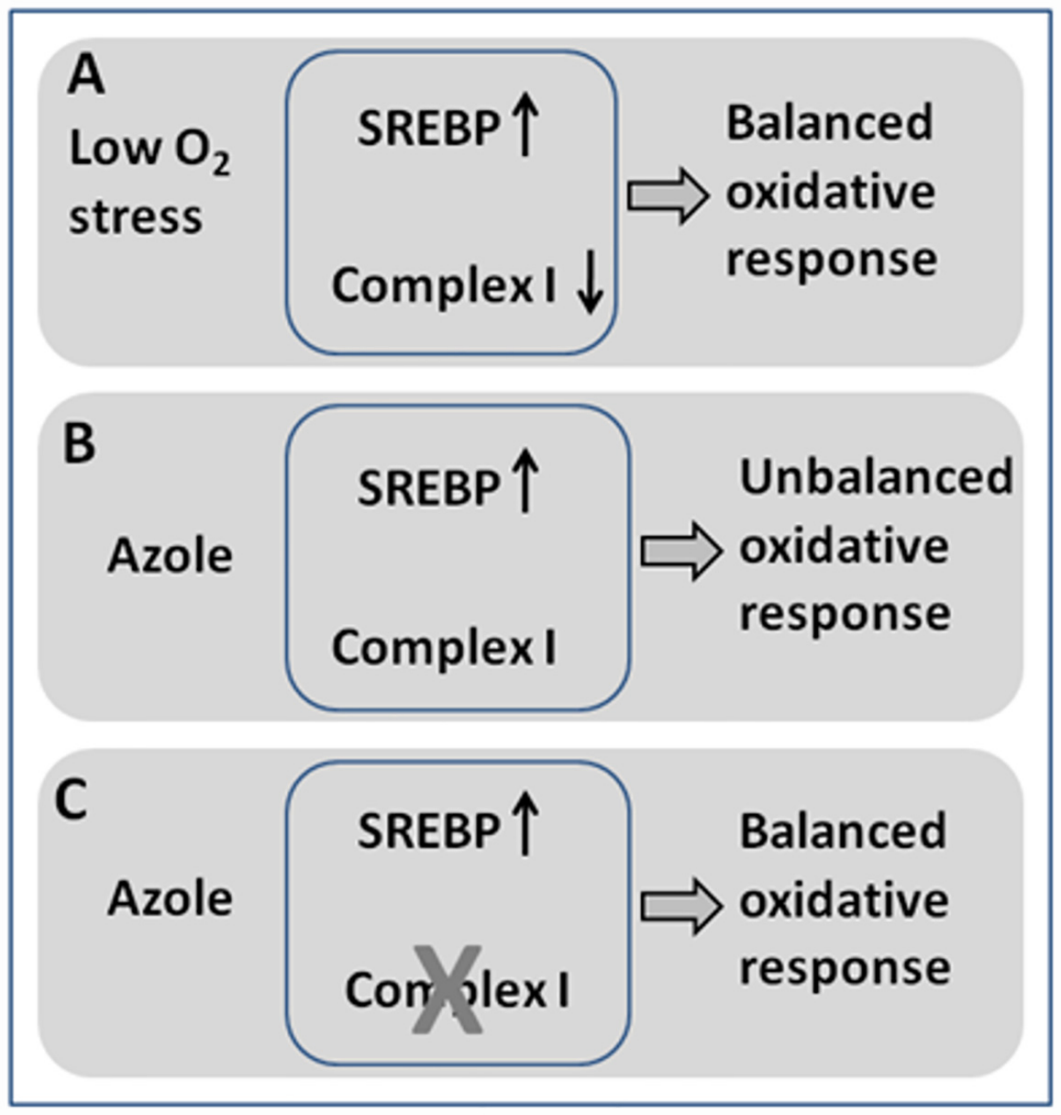
Proposed mechanism for azole resistance via loss of complex I activity. A. In low O_2_ sterol biosynthesis is reduced leading to SREBP signalling and complex I activity is reduced. Both regulatory systems contribute to a balanced oxygen stress response. B Azoles mimic low oxygen by reducing sterol biosynthesis. However oxygen levels are normal and complex I regulation does not occur leading to an unbalanced response. C. Azole reduces sterol biosynthesis but this is balanced by loss of complex I activity due either to rotenone treatment or failure to reactivate the complex because of deletion of the 29.9 KD subunit.

Blockage of the sterol biosynthesis pathway results in deficiency in oxygen sensing via SREBP. This induces a hypoxic response in the presence of normal oxygen levels allowing respiration to remain fully active. This could result in an unbalanced hypoxic response and hence cell death or stasis (Fig 7). Previous work has demonstrated that hypoxia alone does not induce azole resistance in *A*. *fumigatus* and that the SREBP knockout does not appear to regulate mitochondrial respiration. The observed reduction in complex I activity in the Δ29.9 mutant matches that seen in adaptation of the parental strain to hypoxia. Our suggestion is that this could restore the balance of the hypoxic response during azole action with restoration of cell growth. One further possible explanation concerns the proposed function of complex I in resistance to stress such as heat or free radical damage. In this case stress caused by azole action might act synergistically with complex I generation of superoxide so that reduction in complex I activity would reduce cellular redox stress.

Loss of virulence in the 29.9KD knockout might result directly from loss of secondary metabolite production as many secondary metabolites are known to affect host cell responses or are thought to be potential virulence factors. Alternatively loss of metabolic control by deregulation of complex I may affect other virulence functions. Lipid rather than glucose is the carbon source likely to be used by fungi in the lung. Since lipids are metabolised via the glyoxylate cycle and gluconeogenesis in the same manner as acetate the observed differences in complex I activation are likely to play an important role in early stages of lung disease. In this scenario expression of secondary metabolite clusters could be repressed during germination of the 29.9KD knockout in lung surfactant resulting in loss of virulence [68]. A further important consideration is outlined in a recent review by Shingu-Vasquez and Traven [56] who outline the probable role of mitochondria in fungal drug resistance via alteration in memebrane dynamics or composition. Our data (S1 and S3 Tables) do show strong down-regulation of the *A*. *fumigatus* PSD1 (phosphoserine decarboxylase) orthologue (AFUA_6G00260) suggesting possible involvement of membrane composition or dynamics in the observed resistance phenotype.

Since genetic or chemical reduction of complex I activity leads to the observed phenotypes it is reasonable to suggest that complex I forms a key component in regulation of secondary metabolism, virulence and drug resistance. The 29.9KD subunit is not a transcription factor and its localisation in the mitochondria suggests that it is unlikely to function in this manner. Fungi do not possess an orthologue of the mammalian HIFα protein that regulates complex I activity in response to hypoxia. However the clear similarity in regulation of activity in *A*. *fumigatus* and mammalian systems suggest that unknown control mechanisms do exist. The consequences of the multiple phenotypes of complex I inhibition in a clinical setting are unpredictable. Growth on predominantly lipid containing surfactant would be expected to reduce complex I activity in the same manner as growth on acetate and hence increase azole resistance as lipids are metabolised via 2-carbon metabolism. Sensitivity to azole could be resumed when glucose or other hexose carbon sources become available. At the same time growth on lipid might reduce expression of secondary metabolites and virulence.

The observation that secondary metabolite clusters are strongly regulated by itraconazole may have clinical implications. For example genes responsible for the biogeneration of the highly toxic and immunosuppressive metabolite gliotoxin are strongly up-regulated by itraconazole. Patients that are undergoing azole treatment may expect localised immunosuppression around the site of infection antagonising the effect of the drug. Strikingly, our results suggest that monitoring for azole resistance has added importance, as for drug resistantisolates the problem of secondary metabolite induction by azoles will be further exacerbated.

## Supporting Information

S1 Fig Complementation of pPTRII-29.9 in mutant strains. (DOCX)

S2 Fig Complex I gene knockouts mediate azole resistance in *N*. *Crassa*. (DOCX)

S3 Fig Analysis of changes in gene expression for secondary metabolite gene clusters in *A*. *fumigatus*. (DOCX)

S1 Table Genes >4 fold differentially down—regulated in the presence of itraconazole (Δ 29.9KD vs parental). (DOCX)

S2 Table Genes >4 fold differentially up—regulated in the presence of itraconazole (Δ 29.9KD vs parental). (DOCX)

S3 Table Genes >4 fold more highly expressed without addition of itraconazole (Δ 29.9KD vs parental). (DOCX)

S4 Table Genes >4 fold under-expressed without addition of itraconazole (Δ 29.9KD vs parental). (DOCX)

S5 Table Primers used in this study. (DOCX)

S6 Table *N*. *crassa* mutants used in this study. (DOCX)
