# Correction: Mitochondrial Complex I Is a Global Regulator of Secondary Metabolism, Virulence and Azole Sensitivity in Fungi

**DOI:** 10.1371/journal.pone.0171787

**Published:** 2017-02-02

**Authors:** Mike Bromley, Anna Johns, Emma Davies, Marcin Fraczek, Jane Mabey Gilsenan, Natalya Kurbatova, Maria Keays, Misha Kapushesky, Marta Gut, Ivo Gut, David W. Denning, Paul Bowyer

The following information is missing from the Funding section: PB and MB were also funded by MRC grant MR/M02010X/1.
