# Supplementary material for: Mitochondrial Complex I Is a Global Regulator of Secondary Metabolism, Virulence and Azole Sensitivity in Fungi
Source: PLoS One. 2016 Jul 20;11(7):e0158724. doi: 10.1371/journal.pone.0158724 (PMC4954691; doi:10.1371/journal.pone.0158724)
Supplement: S1 Fig — (DOCX) [file pone.0158724.s001.docx]

**Complementation of the AF210:101 REMI mutant using a replicating plasmid containing the 29.9KD gene.**


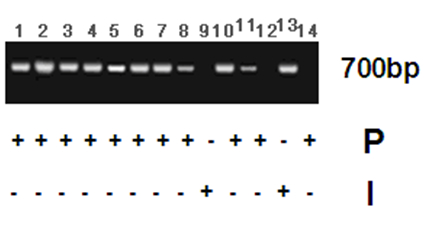


**S1 Fig. Complementation of pPTRII-29.9 in mutant strains.** A replicating plasmid pPTRII containing a pyrithiamine resistance marker and the 29.9KD gene and flanking sequences was used to transform the Af210:101 mutant. Lanes 1-7 show primary pyrithiamine resistant transformants carrying pPTRII-29.9 as assessed by PCR. Lanes 8-14 show transformants that have been sub-cultured without selection. Lanes 9 and 14 did not give a PCR band, are pyrithiamine sensitive and have reverted to azole resistance. P: pyrithiamine resistance is scored positive for transformants able to grow in the presence of 100ng/ml pyrithiamine and negative for colonies that fail to grow at this concentration of pyrithiamine. I: itraconazole resistance is scored as positive for colonies able to grow on 4 mg/l itraconazole (>50% unamended growth) and negative for colonies that are unable to grow (<10% of unamended growth).

The colonies were sub-cultured onto complete medium without pyrithiamine selection and allowed to sporulate. Spores were streaked to single colony and 7 were tested for the presence of the pPTRII-29.9 plasmid as described (Figure 1C, lanes 8-14). Two colonies appeared to have lost the plasmid in the absence of selection (lanes 9 and 14). These colonies were resistant to 4 mg/l itraconazole and appeared to be phenotypically identical to AF210:101. These data strongly suggest that the observed itraconazole resistance in AF210:101 is mediated by loss of function of the 29.9KD subunit of complex I.
