# Supplementary material for: Mitochondrial Complex I Is a Global Regulator of Secondary Metabolism, Virulence and Azole Sensitivity in Fungi
Source: PLoS One. 2016 Jul 20;11(7):e0158724. doi: 10.1371/journal.pone.0158724 (PMC4954691; doi:10.1371/journal.pone.0158724)
Supplement: S2 Fig — (DOCX) [file pone.0158724.s002.docx]

**S2 Fig. Complex I gene knockouts mediate azole resistance in *N. crassa***

In order to further investigate the role of complex I in determining azole resistance it was decided to take advantage of an existing set of complex I gene knockouts previously generated in *N. crassa*. Complex I has been extensively studied in *N. crassa* and functions or putative structural positions for these genes have been assigned in many cases [From NADH to ubiquinone in Neurospora mitochondria[46].

The mutants and wild type were tested for their sensitivity to azoles in the presence or absence of 1mM rotenone by assessing growth rate in race tubes. One group of mutants consisting of knockouts in the NCU09299.2, NCU01142.2, NCU04044.2, NCU02754.2, NCU02814.2, NCU01859.2, NCU01467.2 genes (corresponding to the 16.6KD, 17.2KD, Nuo 51 flavoprotein 1, Nuo49, 21KD, and NUO-14 subunits respectively) were azole resistant in the absence of rotenone (Figure 5A) whereas the remaining mutants did not display azole resistance in the absence of rotenone but showed azole resistance in the presence of rotenone (Figure 5B).


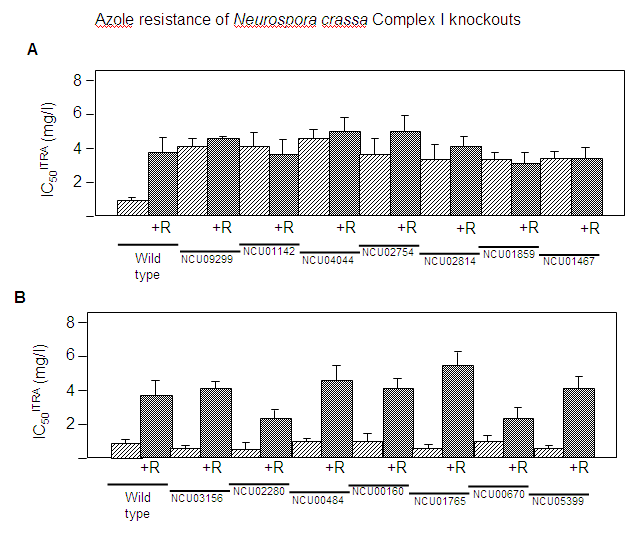


**S2 Fig. Azole resistance of *N. crassa* complex I sub-unit knockouts in the presence and absence of retenone**. IC_50_^ITRA^ is shown for cultures in the presence (+R) or absence of 1mM rotenone. Panel A shows knockout mutants that have intrinsic azole resistance in the absence of rotenone and where azole resistance is not significantly increased in the presence of rotenone. Panel B shows knockouts where IC_50_^ITRA^ is similar to wild type but is significantly increased by addition of rotenone.
