# Supplementary material for: Mitochondrial Complex I Is a Global Regulator of Secondary Metabolism, Virulence and Azole Sensitivity in Fungi
Source: PLoS One. 2016 Jul 20;11(7):e0158724. doi: 10.1371/journal.pone.0158724 (PMC4954691; doi:10.1371/journal.pone.0158724)

**S3 Fig. Analysis of changes in gene expression for secondary metabolite gene clusters in *A. fumigatus*.**

**S3 Fig. Changes in gene expression of secondary metabolite gene clusters comparing the effects of deletion of the 29.9KD gene and addition of itraconazole.** A. Log_2_ fold change in expression of genes in secondary metabolite clusters in the Δ29.9 KD strain relative to the parental strain in the presence of itraconazole. B. Log_2_ fold change in expression of genes in secondary metabolite clusters in the Δ29.9 KD strain relative to the parental strain with no itraconazole. C. Log_2_ fold change in expression of genes in secondary metabolite clusters in the parental strain in the presence of itraconazole relative to expression in the absence of itracona


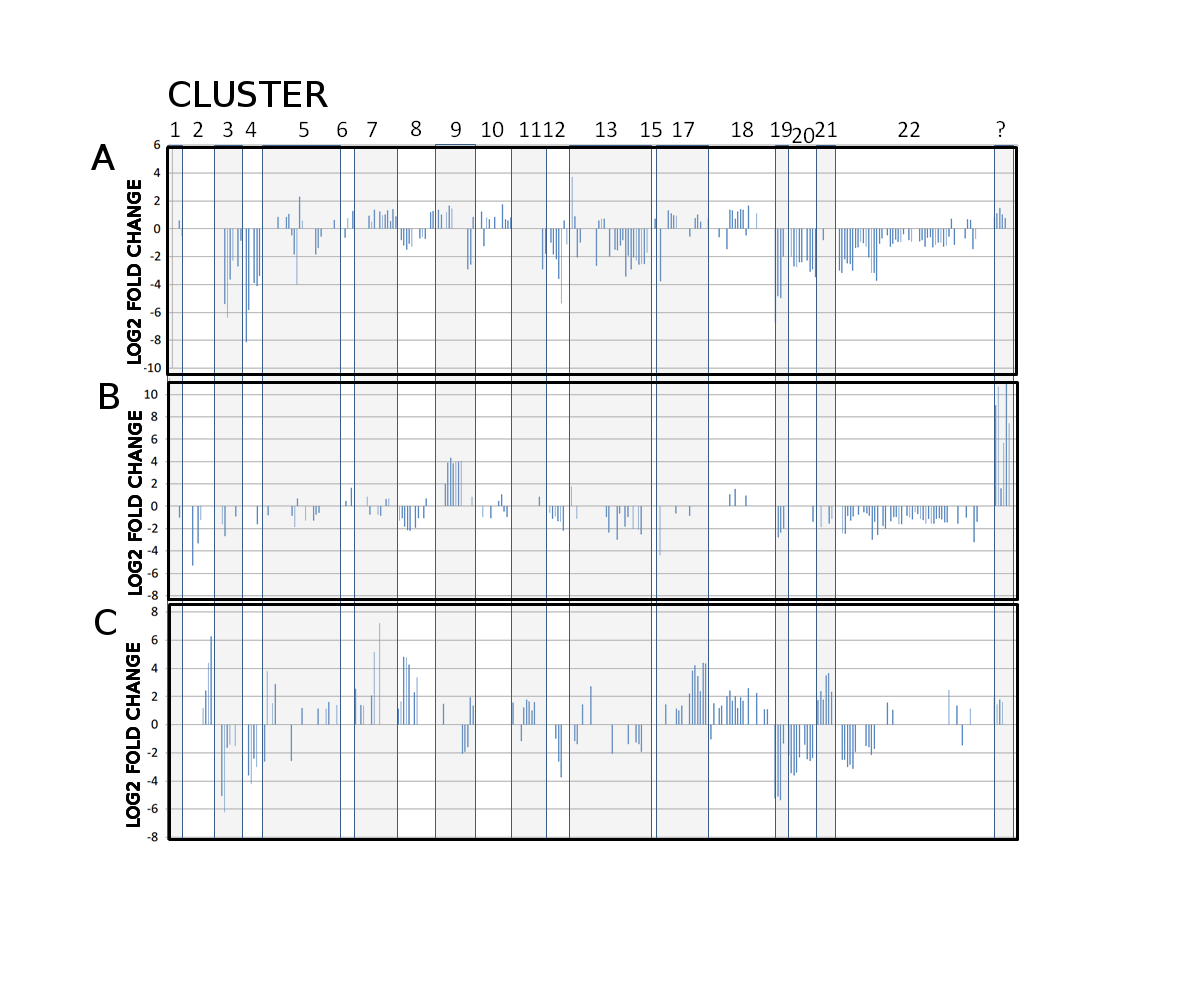

Supplement: S3 Fig — (DOCX) [file pone.0158724.s003.docx]
