# Supplementary material for: Mitochondrial Complex I Is a Global Regulator of Secondary Metabolism, Virulence and Azole Sensitivity in Fungi
Source: PLoS One. 2016 Jul 20;11(7):e0158724. doi: 10.1371/journal.pone.0158724 (PMC4954691; doi:10.1371/journal.pone.0158724)
Supplement: S1 Table — (DOCX) [file pone.0158724.s004.docx]

**S1 Table**.**Genes >4 fold differentially down - regulated in the presence of itraconazole (Δ 29.9KD vs parental).** Genes belonging to secondary metabolite clusters are shaded grey. LogFC; log2 fold change in gene expression, P value: probability of correct assignment from DESEQ, FDR: likelihood offalse discovery rate for the locus from DESEQ, gene ID: gene descriptor from CADRE ([www.cadre.org](http://www.cadre.org)), Cluster ID: secondary metabolism cluster as defined byKhaldi et al. (Khaldi et al Fungal Genet Biol 2010, 47:736-741); note that more up to date assignations of secondary metabolite gene clusters exist (eg Andersen et al. Proc Natl Acad Sci U S A. 2013;110(1):E99-107 or Inglis et al. BMC Microbiol. 2013 Apr 26;13:91) however we chose the older nomenclature for ease of comparison with the laeA analysis.

| **logFC** | **PValue** | **FDR** | **gene_id** | **description** | **Cluster ID** |
| --- | --- | --- | --- | --- | --- |
| **-11.21278074** | **1.66E-242** | **1.49E-238** | **AFUA_2G10600** | **NADH-DH 299 kDa subunit, (1.6.5.3)** |  |
| **-5.813205728** | **5.43E-90** | **2.43E-86** | **AFUA_7G01000** | **aldehyde dehydrogenase, (1.2.1.3)** |  |
| **-5.520255314** | **1.83E-71** | **5.45E-68** | **AFUA_7G01010** | **alcohol dehydrogenase, (1.1.1.1)** |  |
| **-5.455082072** | **2.63E-31** | **2.94E-28** | **AFUA_7G00990** | **AlcS** |  |
| **-4.387974956** | **2.28E-18** | **4.64E-16** | **AFUA_6G03290** | **isochorismatase family hydrolase, (1.1.1.-)** | **19** |
| **-3.870102599** | **8.11E-18** | **1.48E-15** | **AFUA_6G00260** | **phosphatidylserine decarboxylase, (4.1.1.-)** |  |
| **-3.307827314** | **8.89E-15** | **9.15E-13** | **AFUA_1G17680** | **MFS transporter, (similar to HOL1)** |  |
| **-3.207334682** | **3.29E-22** | **1.28E-19** | **AFUA_8G00710** | **secreted antimicrobial peptide AMP3** | **24** |
| **-3.086091075** | **6.44E-10** | **3.41E-08** | **AFUA_3G01100** | **C2H2 finger domain protein** |  |
| **-3.049479937** | **1.61E-21** | **5.78E-19** | **AFUA_6G14000** | **toxin biosynthesis protein Tri7-like** | **22** |
| **-3.00605575** | **3.56E-30** | **3.54E-27** | **AFUA_8G00230** | **Alpha-ketoglutarate dioxygenase** | **24** |
| **-3.004291196** | **3.46E-09** | **1.50E-07** | **AFUA_4G14712** | **C6 transcription factor** | **15** |
| **-2.830745597** | **1.80E-19** | **5.04E-17** | **AFUA_8G06520** | **peptidyl-arginine deiminase** |  |
| **-2.804913844** | **1.85E-10** | **1.08E-08** | **AFUA_6G12050** | **nonribosomal peptide synthase, (6.3.2.-)** | **21** |
| **-2.799532339** | **3.22E-19** | **8.48E-17** | **AFUA_3G15050** | **flavin-binding monooxygenase, putative** |  |
| **-2.779462173** | **1.63E-16** | **2.36E-14** | **AFUA_2G01280** | **D-mandelate dehydrogenase, (1.1.1.-)** |  |
| **-2.764964217** | **1.86E-08** | **7.12E-07** | **AFUA_8G06030** | **alpha-1,3-glucanase, MutA (3.2.1.-)** |  |
| **-2.709435294** | **2.96E-10** | **1.66E-08** | **AFUA_5G14740** | **Fucose-specific lectin** |  |
| **-2.678957392** | **5.06E-07** | **1.39E-05** | **AFUA_2G17540** | **Brown 1** | **3** |
| **-2.612941192** | **2.89E-10** | **1.64E-08** | **AFUA_8G02510** | **glycosyl hydrolase family 43 protein** |  |
| **-2.593567453** | **1.84E-16** | **2.61E-14** | **AFUA_8G00250** | **Prenyltransferase** | **24** |
| **-2.56265849** | **1.14E-35** | **2.54E-32** | **AFUA_5G07500** | **beta-lactamase family protein, LovD** |  |
| **-2.522962682** | **6.94E-08** | **2.34E-06** | **AFUA_7G01070** | **endo-1,4-beta-mannosidase** |  |
| **-2.509950415** | **4.45E-06** | **9.68E-05** | **AFUA_4G14830** | **cytochromeP450 monooxygenase, (1.14.-.)** | **15** |
| **-2.488697107** | **1.02E-25** | **5.38E-23** | **AFUA_8G00110** | **2OG-Fe(II) oxygenase family,** | **24** |
| **-2.441267194** | **9.55E-33** | **1.71E-29** | **AFUA_6G08760** | **proline oxidase PrnD** |  |
| **-2.400882192** | **4.27E-19** | **1.06E-16** | **AFUA_2G10230** | **inositol oxygenase, (1.13.99.1)** |  |
| **-2.386362925** | **4.64E-30** | **4.16E-27** | **AFUA_8G00100** | **aspartate-tRNA ligase,(6.1.1.12)** | **24** |
| **-2.370035826** | **7.95E-12** | **5.47E-10** | **AFUA_6G12060** | **Maackiain detoxification protein 1** | **21** |
| **-2.364786009** | **4.80E-21** | **1.53E-18** | **AFUA_7G01090** | **proline permease PrnB** |  |
| **-2.350384294** | **2.91E-17** | **4.56E-15** | **AFUA_4G14670** | **MFS quinate permease** | **15** |
| **-2.338101383** | **9.54E-17** | **1.40E-14** | **AFUA_1G17320** | **endo-arabinanase, putative** |  |
| **-2.277730452** | **2.21E-09** | **1.01E-07** | **AFUA_8G00930** | **chitosanase, putative, (3.2.1.132)** |  |
| **-2.267229123** | **1.53E-09** | **7.25E-08** | **AFUA_2G17895** | **C6 transcription factor** |  |
| **-2.250371271** | **6.74E-28** | **4.31E-25** | **AFUA_5G01030** | **glyceraldehyde 3-phosphate dehydrogenase** |  |
| **-2.244198355** | **0.006051** | **0.0422121** | **AFUA_8G06690** | **cytochrome P450 alkane hydroxylase** |  |
| **-2.21162385** | **4.78E-19** | **1.16E-16** | **AFUA_3G00640** | **peptidoglycan binding domain protein** |  |
| **-2.199341905** | **1.68E-28** | **1.16E-25** | **AFUA_8G00920** | **UDP-glucose dehydrogenase Ugd1** |  |
| **-2.1953774** | **1.13E-10** | **6.80E-09** | **AFUA_4G00230** | **oxidoreductase, 2OG-Fe(II) oxygenase family** | **14** |
| **-2.189723846** | **2.34E-22** | **9.51E-20** | **AFUA_3G02175** | **BTB/POZ domain protein** |  |
| **-2.185756218** | **1.00E-18** | **2.30E-16** | **AFUA_3G12920** | **nonribosomal peptide synthase GliP-like,(6.3.2.-)** | **10** |
| **-2.179001716** | **2.95E-12** | **2.14E-10** | **AFUA_2G00710** | **alpha-amylase, putative AmyB (3.2.1.1)** |  |
| **-2.164060681** | **7.89E-18** | **1.47E-15** | **AFUA_1G17170** | **TfdA taurine dioxygenase, (1.14.11.-)** |  |
| **-2.163997826** | **1.50E-12** | **1.15E-10** | **AFUA_4G04318** | **copper resistance protein Crd2** |  |
| **-2.16291315** | **4.40E-14** | **4.33E-12** | **AFUA_3G12910** | **O-methyltransferase GliM-like SirM (2.-** | **10** |
| **-2.146195175** | **5.84E-15** | **6.22E-13** | **AFUA_6G13990** | **C-8 acyltransferase** | **22** |
| **-2.130761927** | **3.48E-16** | **4.72E-14** | **AFUA_5G01230** | **RTA1 domain protein** |  |
| **-2.115874332** | **1.62E-06** | **3.99E-05** | **AFUA_8G02150** | **acetylornithine aminotransferase (2.6.1.11)** |  |
| **-2.09361839** | **6.08E-15** | **6.40E-13** | **AFUA_5G07490** | **cyclopentanone 1,2-monooxygenase CpmA** |  |
| **-2.078842399** | **0.000584** | **0.0065177** | **AFUA_4G14820** | **hydroxyanthranilate hydroxycinnamoyl transferase** | **15** |
| **-2.053635895** | **0.00237** | **0.0203237** | **AFUA_4G14800** | **short chain dehydrogenase, (1.1.1.-)** | **15** |
| **-2.044635949** | **5.88E-10** | **3.13E-08** | **AFUA_2G14520** | **hydrolase, putative, (3.-.-.-)** | **3** |
| **-2.028276144** | **1.95E-05** | **0.0003617** | **AFUA_2G03960** | **acid phosphatase, (3.1.3.2)** |  |
| **-2.012159383** | **4.51E-09** | **1.87E-07** | **AFUA_8G06680** | **acyl-CoA thioesterase, (3.1.2.-)** |  |
| **-2.001910109** | **8.91E-26** | **4.99E-23** | **AFUA_1G17250** | **Conidial hydrophobin RodB** |  |
