# Supplementary material for: Mitochondrial Complex I Is a Global Regulator of Secondary Metabolism, Virulence and Azole Sensitivity in Fungi
Source: PLoS One. 2016 Jul 20;11(7):e0158724. doi: 10.1371/journal.pone.0158724 (PMC4954691; doi:10.1371/journal.pone.0158724)
Supplement: S2 Table — (DOCX) [file pone.0158724.s005.docx]

**S2 Table. Genes >4 fold differentially up - regulated in the presence of itraconazole (Δ 29.9KD vs parental).** Genes belonging to secondary metabolite clusters are shaded grey.

| LogFC | P value | FDR | Gene ID | Description | Cluster ID |
| --- | --- | --- | --- | --- | --- |
| **2.0568576242** | **1.31E-009** | **6.40E-008** | **AFUA_6G02810** | **Ctr copper transporter, putative** |  |
| **2.0585087578** | **2.13E-007** | **6.49E-006** | **AFUA_3G13640** | **extracellular serine-rich protein, putative** | **11** |
| **2.111983825** | **3.75E-019** | **9.59E-017** | **AFUA_3G06720** | **ThiJ/PfpI family protein, contains a ThiJ/PfpI domain** |  |
| **2.4187959393** | **4.35E-009** | **1.82E-007** | **AFUA_4G03940** | **Ferric reductase transmembrane component 2 (1.16.1.7)** |  |
| **2.4762088007** | **1.12E-009** | **5.59E-008** | **AFUA_6G13750** | **ferric-chelate reductase, putative, (1.16.1.7)** |  |
| **3.8792236802** | **5.02E-020** | **1.55E-017** | **AFUA_3G13670** | **siderochrome-iron transporter, putative** | **11** |
| **3.8860257269** | **6.30E-016** | **8.17E-014** | **AFUA_3G13650** | **integral membrane protein, (1.1.3.25)** | **11** |
| **3.9712670002** | **6.40E-014** | **6.16E-012** | **AFUA_3G13690** | **pyoverdine/dityrosine biosynthesis protein** | **11** |
| **4.0139239277** | **1.11E-023** | **5.23E-021** | **AFUA_3G13680** | **conserved hypothetical protein** | **11** |
| **4.0418704362** | **3.10E-018** | **6.04E-016** | **AFUA_3G13700** | **Transferase, trichothecene 3-O-acetyltransferase** | **11** |
| **4.3526626284** | **6.49E-024** | **3.23E-021** | **AFUA_3G13660** | **low-affinity copper transporter** | **11** |
| **4.6128550137** | **2.26E-017** | **3.61E-015** | **AFUA_8G01310** | **ferric-chelate reductase (Fre2p), (1.16.1.-)** |  |
