# Supplementary material for: Mitochondrial Complex I Is a Global Regulator of Secondary Metabolism, Virulence and Azole Sensitivity in Fungi
Source: PLoS One. 2016 Jul 20;11(7):e0158724. doi: 10.1371/journal.pone.0158724 (PMC4954691; doi:10.1371/journal.pone.0158724)
Supplement: S3 Table — (DOCX) [file pone.0158724.s006.docx]

**S3 Table. Genes >4 fold more highly expressed without addition of itraconazole (Δ 29.9KD vs parental).** Genes belonging to secondary metabolite clusters are shaded grey.

| **Log FC** | **P value** | **FDR** | **Gene ID** | **Description** | **Cluster ID** |
| --- | --- | --- | --- | --- | --- |
| **3.715710206** | **3.29E-83** | **1.64E-80** | **AFUA_4G14380** | **glutathione S-transferase** | **15** |
| **3.543547316** | **0.0007892** | **0.004306** | **AFUA_5G10150** | **hypothetical protein** |  |
| **3.461874839** | **1.21E-07** | **1.44E-06** | **AFUA_2G17930** | **Sterol desaturase/short chain dehydrogenase** |  |
| **3.33866364** | **1.17E-54** | **1.84E-52** | **AFUA_2G17300** | **glutathione S-transferase** |  |
| **3.283201573** | **1.16E-74** | **4.71E-72** | **AFUA_5G06070** | **Multidrug resistance protein 1** |  |
| **3.186355226** | **3.16E-06** | **2.96E-05** | **AFUA_4G00710** | **C6 transcription factor** |  |
| **2.950840521** | **6.23E-20** | **2.50E-18** | **AFUA_1G03352** | **alpha-1,3-glucanase/mutanase (3.2.1.-)** |  |
| **2.746447748** | **7.97E-07** | **8.37E-06** | **AFUA_7G06200** | **ankyrin repeat protein** |  |
| **2.694182758** | **1.67E-05** | **0.0001376** | **AFUA_6G03080** | **ABC multidrug transporter** |  |
| **2.563935451** | **7.52E-62** | **1.53E-59** | **AFUA_1G12620** | **AflT-like MFS transporter** |  |
| **2.359049645** | **1.77E-23** | **8.39E-22** | **AFUA_8G01400** | **mitochondrial carrier protein (Pet8), putative** |  |
| **2.318086808** | **6.35E-39** | **5.92E-37** | **AFUA_6G07670** | **cytochrome c oxidase assembly cox15** |  |
| **2.310934861** | **1.48E-43** | **1.64E-41** | **AFUA_3G01400** | **ABC multidrug transporter, (3.6.3.-)** | **6** |
| **2.249528903** | **1.83E-44** | **2.07E-42** | **AFUA_1G10750** | **prolyl-tRNA synthetase, (6.1.1.15)** |  |
| **2.208051557** | **2.10E-11** | **4.11E-10** | **AFUA_4G03700** | **GMP synthase, (6.3.5.2)** |  |
| **2.146886091** | **2.21E-38** | **2.04E-36** | **AFUA_1G03800** | **C6 transcription factor, putative** |  |
| **2.103674255** | **5.62E-48** | **7.74E-46** | **AFUA_6G09310** | **class V chitinase, ChiC (3.2.1.14)** |  |
| **2.096469882** | **8.22E-07** | **8.59E-06** | **AFUA_8G01250** | **GNAT family acetyltransferase, putative** |  |
| **2.09180589** | **1.36E-32** | **9.89E-31** | **AFUA_3G07300** | **ABC multidrug transporter, (3.6.3.-)** |  |
| **2.069360389** | **1.08E-31** | **7.70E-30** | **AFUA_2G16860** | **MFS multidrug transporter** |  |
| **2.02821505** | **5.35E-12** | **1.12E-10** | **AFUA_5G01620** | **extracellular proline-rich protein** |  |
|  |  |  |  |  |  |
