# Supplementary material for: Mitochondrial Complex I Is a Global Regulator of Secondary Metabolism, Virulence and Azole Sensitivity in Fungi
Source: PLoS One. 2016 Jul 20;11(7):e0158724. doi: 10.1371/journal.pone.0158724 (PMC4954691; doi:10.1371/journal.pone.0158724)
Supplement: S4 Table — (DOCX) [file pone.0158724.s007.docx]

**S4 Table. Genes >4 fold under-expressed without addition of itraconazole (Δ 29.9KD vs parental).** Genes belonging to secondary metabolite clusters are shaded grey.

| **logFC** | **PValue** | **FDR** | **Gene ID** | **Description** | Cluster ID |
| --- | --- | --- | --- | --- | --- |
| **-10.96610457** | **0** | **0** | **AFUA_2G10600** | **NADH- DH 29.9 kDa subunit,(1.6.5.3)** |  |
| **-8.14593637** | **3.78E-58** | **6.90E-56** | **AFUA_2G17960** | **NADPH dehydrogenase Oye3, (1.6.99.1)** | **5** |
| **-6.717812376** | **1.04E-181** | **3.11E-178** | **AFUA_6G12050** | **nonribosomal peptide synthase (6.3.2.-)** | **21** |
| **-6.409945833** | **6.33E-23** | **2.94E-21** | **AFUA_8G06690** | **cytochrome P450 alkane hydroxylase** |  |
| **-6.398208959** | **2.89E-163** | **6.47E-160** | **AFUA_2G17540** | **Brown 1** | **4** |
| **-5.808629962** | **9.14E-46** | **1.12E-43** | **AFUA_2G17970** | **ergot alkaloid biosynthetic protein A** | **5** |
| **-5.390878641** | **2.35E-91** | **1.40E-88** | **AFUA_2G17530** | **Brown 2** | **4** |
| **-5.338822557** | **1.34E-121** | **1.71E-118** | **AFUA_4G00230** | **2OG-Fe(II) oxygenase family,** | **14** |
| **-4.953759674** | **8.55E-183** | **3.83E-179** | **AFUA_6G12070** | **FAD binding domain protein (1.5.3.-)** | **21** |
| **-4.843894672** | **3.76E-137** | **6.74E-134** | **AFUA_6G12060** | **maackiain detoxification protein 1 ((EC 1.14.13.1)** | **21** |
| **-4.557692478** | **2.10E-34** | **1.65E-32** | **AFUA_2G00840** | **MFS transporter** |  |
| **-4.546602001** | **3.32E-46** | **4.19E-44** | **AFUA_3G01100** | **C2H2 finger domain protein, putative** | **?** |
| **-4.175343285** | **8.01E-119** | **8.96E-116** | **AFUA_2G14750** | **endo-arabinase, putative** |  |
| **-4.157452928** | **1.80E-45** | **2.18E-43** | **AFUA_2G01280** | **D-mandelate dehydrogenase, (1.1.1.-)** | **3** |
| **-4.120216994** | **3.53E-20** | **1.43E-18** | **AFUA_2G18000** | **short chain oxidoreductase CpoX2,** | **5** |
| **-4.008320354** | **6.43E-66** | **1.92E-63** | **AFUA_8G06680** | **peroxisomal acyl-CoA thioesterase (3.1.2.-)** |  |
| **-3.995334513** | **2.44E-102** | **1.82E-99** | **AFUA_5G08180** | **cell wall protein, putative** |  |
| **-3.976475569** | **1.58E-18** | **5.93E-17** | **AFUA_3G01370** | **MFS transporter** | **6** |
| **-3.903400836** | **9.55E-18** | **3.29E-16** | **AFUA_2G17990** | **dimethylallyl tryptophan synthase FgaPT1** | **5** |
| **-3.761153274** | **3.17E-34** | **2.45E-32** | **AFUA_6G03290** | **isochorismatase family hydrolase (1.1.1.-)** | **19** |
| **-3.74372607** | **7.02E-66** | **2.03E-63** | **AFUA_8G00250** | **Prenyltransferase** | **24** |
| **-3.693974255** | **2.39E-37** | **2.02E-35** | **AFUA_4G03240** | **Antigenic cell wall galactomannoprotein** |  |
| **-3.682907613** | **4.60E-43** | **4.90E-41** | **AFUA_6G00260** | **phosphatidylserine decarboxylase (4.1.1.-)** |  |
| **-3.642801191** | **1.57E-42** | **1.60E-40** | **AFUA_2G17550** | **Yellowish-green 1** | **4** |
| **-3.552582242** | **8.19E-13** | **1.82E-11** | **AFUA_3G01230** | **MFS sugar transporter** | **?** |
| **-3.509973017** | **3.64E-110** | **3.26E-107** | **AFUA_6G00770** | **extracellular arabinanase** |  |
| **-3.489678213** | **6.62E-58** | **1.18E-55** | **AFUA_8G06030** | **alpha-1,3-glucanase, MutA (3.2.1.-)** |  |
| **-3.488673777** | **1.22E-84** | **6.41E-82** | **AFUA_6G14000** | **Toxin biosynthesis protein Tri7-like** | **22** |
| **-3.418605898** | **2.47E-13** | **5.79E-12** | **AFUA_4G14770** | **Lanosterol synthase (5.4.99.7)** | **15** |
| **-3.390061162** | **2.44E-60** | **4.85E-58** | **AFUA_2G18030** | **catalase (1.11.1.6)** | **5** |
| **-3.340630803** | **6.32E-44** | **7.06E-42** | **AFUA_8G06520** | **peptidyl-arginine deiminase domain protein,** |  |
| **-3.266594989** | **1.75E-82** | **8.24E-80** | **AFUA_6G07855** | **conserved threonine rich protein** |  |
| **-3.213221143** | **1.10E-26** | **6.15E-25** | **AFUA_7G00990** | **AlcS** |  |
| **-3.180267788** | **2.31E-64** | **5.17E-62** | **AFUA_8G00230** | **Alpha-ketoglutarate dioxygenase** | **24** |
| **-3.173794738** | **6.67E-42** | **6.71E-40** | **AFUA_8G00240** | **Cytochrome P450** | **24** |
| **-3.1727462** | **1.03E-81** | **4.61E-79** | **AFUA_8G00110** | **oxidoreductase, 2OG-Fe(II) oxygenase family,** | **24** |
| **-3.171018515** | **7.51E-59** | **1.40E-56** | **AFUA_4G09830** | **indoleamine 2,3-dioxygenase** |  |
| **-3.12532867** | **4.15E-65** | **1.00E-62** | **AFUA_1G17250** | **Conidial hydrophobin RodB** |  |
| **-3.076682335** | **1.35E-65** | **3.77E-63** | **AFUA_6G13980** | **UbiA-like prenyltransferase** | **22** |
| **-3.017550036** | **1.60E-30** | **1.07E-28** | **AFUA_8G00150** | **conserved hypothetical protein** | **24** |
| **-2.982708157** | **1.34E-94** | **9.21E-92** | **AFUA_8G00100** | **aspartate-tRNA ligase, (6.1.1.12)** | **24** |
| **-2.974710134** | **3.92E-31** | **2.70E-29** | **AFUA_2G17160** | **25D9-9** |  |
| **-2.95300092** | **2.43E-93** | **1.55E-90** | **AFUA_5G15060** | **terpene synthase family protein** |  |
| **-2.928333652** | **3.48E-69** | **1.11E-66** | **AFUA_6G13990** | **C-8 acyltransferase** | **22** |
| **-2.922194471** | **2.57E-18** | **9.34E-17** | **AFUA_3G13720** | **2OG-Fe(II) oxygenase family** | **11** |
| **-2.920641367** | **9.23E-18** | **3.19E-16** | **AFUA_4G14790** | **cytochrome P450 monooxygenase, (1.14.-.-)** | **15** |
| **-2.916819828** | **4.78E-63** | **1.02E-60** | **AFUA_3G03700** | **MFS sugar transporter** |  |
| **-2.900670978** | **2.97E-25** | **1.57E-23** | **AFUA_2G10230** | **inositol oxygenase (1.13.99.1)** |  |
| **-2.899396152** | **5.96E-32** | **4.26E-30** | **AFUA_3G15290** | **C6 transcription factor** | **13** |
| **-2.890984115** | **7.86E-14** | **1.95E-12** | **AFUA_4G03280** | **short-chain dehydrogenase family** |  |
| **-2.874488181** | **4.82E-74** | **1.80E-71** | **AFUA_4G06880** | **HLH DNA binding domain protein** |  |
| **-2.830959348** | **3.91E-32** | **2.82E-30** | **AFUA_8G01790** | **integral membrane protein** |  |
| **-2.809463944** | **1.11E-29** | **7.02E-28** | **AFUA_3G11490** | **GABA permease, putative** |  |
| **-2.794230783** | **5.89E-43** | **6.13E-41** | **AFUA_1G01812** | **MFS transporter** |  |
| **-2.764364966** | **5.35E-60** | **1.02E-57** | **AFUA_1G16250** | **alpha-glucosidase** |  |
| **-2.756647203** | **1.07E-14** | **2.89E-13** | **AFUA_8G01970** | **extracellular endo-polygalacturonase, (3.2.1.-)** |  |
| **-2.752942346** | **2.16E-33** | **1.62E-31** | **AFUA_7G06140** | **beta-D-glucoside glucohydrolase, (3.2.1.21)** |  |
| **-2.744805685** | **2.18E-78** | **9.30E-76** | **AFUA_6G13940** | **cytochrome P450 monooxygenase** | **22** |
| **-2.706503079** | **1.21E-69** | **4.00E-67** | **AFUA_6G13930** | **LovB-like polyketide synthase** | **22** |
| **-2.705526983** | **3.03E-65** | **7.97E-63** | **AFUA_5G12510** | **adenylate-forming enzyme AfeA** |  |
| **-2.685264193** | **2.00E-47** | **2.63E-45** | **AFUA_2G17580** | **scytalone dehydratase (EC 4.2.1.94)** | **4** |
| **-2.667483761** | **9.25E-06** | **8.00E-05** | **AFUA_3G15060** | **glyoxalase family protein** |  |
| **-2.667258861** | **2.18E-87** | **1.22E-84** | **AFUA_1G15670** | **laccase TilA, (1.10.3.2)** |  |
| **-2.667113622** | **7.80E-22** | **3.42E-20** | **AFUA_4G14530** | **Glutathione transferase 2** | **15** |
| **-2.66545227** | **1.36E-62** | **2.83E-60** | **AFUA_5G12690** | **dihydroxyacetone kinase (DakA), (2.7.1.29)** |  |
| **-2.664169226** | **1.06E-64** | **2.43E-62** | **AFUA_1G05770** | **beta-glucosidase, putative, (3.2.1.21)** |  |
| **-2.655870168** | **1.41E-37** | **1.23E-35** | **AFUA_3G00640** | **peptidoglycan binding domain** |  |
| **-2.639248914** | **3.72E-60** | **7.24E-58** | **AFUA_2G14661** | **hydrophobin, putative,** |  |
| **-2.638081493** | **6.01E-74** | **2.15E-71** | **AFUA_5G15050** | **trichodiene oxygenase, cytochrome P450** |  |
| **-2.573479281** | **1.68E-26** | **9.30E-25** | **AFUA_3G13730** | **nonribosomal peptide synthase,** | **11** |
| **-2.569900566** | **2.32E-10** | **3.97E-09** | **AFUA_4G14820** | **hydroxyanthranilate hydroxycinnamoyl transferase 3** | **15** |
| **-2.564406149** | **4.22E-15** | **1.18E-13** | **AFUA_6G00760** | **glutathione S-transferase,(2.5.1.18)** |  |
| **-2.549933105** | **6.87E-13** | **1.54E-11** | **AFUA_4G14830** | **cytochrome P450 monooxygenase, (1.14.-.-)** | **15** |
| **-2.549089826** | **1.57E-63** | **3.42E-61** | **AFUA_5G14860** | **cytochrome P450** |  |
| **-2.529227264** | **1.10E-06** | **1.12E-05** | **AFUA_4G14840** | **transferase taxadienol acetyl transferase** | **15** |
| **-2.524342109** | **6.22E-51** | **9.28E-49** | **AFUA_8G00140** | **MFS transporter** | **24** |
| **-2.520998157** | **5.51E-43** | **5.81E-41** | **AFUA_5G01240** | **general amidase** |  |
| **-2.491371015** | **2.41E-36** | **1.98E-34** | **AFUA_5G08900** | **D-arabinitol dehydrogenase ArbD, (1.1.1.69)** |  |
| **-2.469473353** | **1.03E-40** | **1.00E-38** | **AFUA_5G01580** | **short chain dehydrogenase/reductase, (1.1.1.100)** |  |
| **-2.441469825** | **1.29E-47** | **1.73E-45** | **AFUA_6G03870** | **tetratricopeptide repeat protein** |  |
| **-2.423381543** | **3.04E-09** | **4.53E-08** | **AFUA_7G05160** | **fumarylacetoacetate hydrolase family** |  |
| **-2.408732535** | **4.10E-65** | **1.00E-62** | **AFUA_6G13950** | **integral membrane protein** | **22** |
| **-2.391101577** | **8.21E-56** | **1.44E-53** | **AFUA_6G13945** | **Cytochrome P450 24A1 (Vitamin D(3) 24-hydroxylase)** | **22** |
| **-2.359523664** | **2.33E-34** | **1.81E-32** | **AFUA_8G06405** | **isoflavone reductase family protein, CipA** |  |
| **-2.349085871** | **1.50E-37** | **1.29E-35** | **AFUA_6G07260** | **purine-cytosine permease** |  |
| **-2.33983332** | **1.15E-51** | **1.75E-49** | **AFUA_1G15490** | **MFS multidrug transporter** |  |
| **-2.339511338** | **6.17E-11** | **1.14E-09** | **AFUA_8G00930** | **chitosanase, putative, (3.2.1.132)** | **24** |
| **-2.332208627** | **2.31E-55** | **3.77E-53** | **AFUA_4G10130** | **alpha-amylase, (3.2.1.1)** |  |
| **-2.3068839** | **1.21E-24** | **6.17E-23** | **AFUA_5G06290** | **MFS transporter, putative** |  |
| **-2.304151925** | **3.82E-43** | **4.12E-41** | **AFUA_7G06380** | **maltase, (3.2.1.70)** |  |
| **-2.290991806** | **3.84E-55** | **6.14E-53** | **AFUA_6G13970** | **FAD-dependent monooxygenase (PaxM), (1.14.13.1)** | **22** |
| **-2.287109102** | **4.59E-09** | **6.64E-08** | **AFUA_8G06670** | **short-chain dehydrogenase/reductase family protein** |  |
| **-2.285164619** | **2.24E-07** | **2.56E-06** | **AFUA_4G14810** | **cytochrome P450 monooxygenase, (1.14.-.-)** | **15** |
| **-2.263900613** | **2.41E-43** | **2.63E-41** | **AFUA_5G01590** | **geranylgeranyl pyrophosphate synthase, (2.5.1.-)** |  |
| **-2.260468559** | **2.50E-23** | **1.17E-21** | **AFUA_2G17560** | **1,3,6,8-tetrahydroxynaphthalene reductase Arp2 ;** | **4** |
| **-2.240178248** | **1.39E-41** | **1.38E-39** | **AFUA_2G11270** | **alpha-1,3-glucan synthase** |  |
| **-2.211285889** | **2.48E-45** | **2.92E-43** | **AFUA_3G15090** | **adenosine deaminase family protein, (3.5.4.4)** |  |
| **-2.208424718** | **3.33E-28** | **1.99E-26** | **AFUA_8G01780** | **nitrilase,** |  |
| **-2.203065746** | **6.74E-20** | **2.69E-18** | **AFUA_4G00220** | **metallo-beta-lactamase domain protein** | **14** |
| **-2.195204717** | **8.56E-48** | **1.16E-45** | **AFUA_1G16590** | **Transcription factor for asexual differentiation** |  |
| **-2.194167653** | **6.40E-38** | **5.67E-36** | **AFUA_6G13270** | **exo-beta-1,3-glucanase, (3.2.1.58)** |  |
| **-2.189247846** | **1.19E-27** | **7.02E-26** | **AFUA_1G05290** | **endo-1,3(4)-beta-glucanase, (3.2.1.6)** |  |
| **-2.168502649** | **1.69E-12** | **3.67E-11** | **AFUA_5G01600** | **PHP domain protein** |  |
| **-2.161425757** | **5.84E-49** | **8.43E-47** | **AFUA_3G01200** | **integral membrane protein Pth11-like** | **?** |
| **-2.161143194** | **8.67E-09** | **1.22E-07** | **AFUA_4G08870** | **choline transport protein** |  |
| **-2.084944175** | **3.06E-36** | **2.49E-34** | **AFUA_7G06390** | **MFS maltose permease MalP,** |  |
| **-2.08019768** | **1.16E-10** | **2.07E-09** | **AFUA_6G11810** | **RTA1 domain protein, putative** |  |
| **-2.071843351** | **2.78E-31** | **1.94E-29** | **AFUA_5G09720** | **flavin containing polyamine oxidase, (1.5.3.-)** |  |
| **-2.048287247** | **2.25E-38** | **2.05E-36** | **AFUA_8G00220** | **Cytochrome P450 (EC 1.14.-.-)** | **24** |
| **-2.048185734** | **6.66E-17** | **2.17E-15** | **AFUA_4G14440** | **NRPS-like enzyme** | **15** |
| **-2.04790724** | **6.60E-05** | **0.000479** | **AFUA_6G02990** | **short chain dehydrogenase/reductase,** |  |
| **-2.046721496** | **3.11E-26** | **1.71E-24** | **AFUA_4G10150** | **alpha-glucosidase AgdA, (3.2.1.20)** |  |
| **-2.040575372** | **2.80E-06** | **2.65E-05** | **AFUA_4G14800** | **short chain dehydrogenase (1.1.1.-)** | **15** |
| **-2.037351207** | **1.07E-14** | **2.88E-13** | **AFUA_4G14670** | **MFS quinate transporter** | **15** |
| **-2.030006258** | **2.21E-48** | **3.14E-46** | **AFUA_3G12200** | **small oligopeptide transporter, OPT family** |  |
| **-2.020898764** | **8.22E-35** | **6.51E-33** | **AFUA_6G12080** | **nonribosomal peptide synthase (6.3.2.-)** | **21** |
| **-2.017485407** | **6.19E-38** | **5.54E-36** | **AFUA_4G10090** | **GABA permease** |  |
| **-2.01113104** | **5.51E-27** | **3.16E-25** | **AFUA_8G01370** | **CmcJ-like methyltransferase** |  |
| **-2.005395655** | **3.36E-30** | **2.20E-28** | **AFUA_3G07030** | **glutaminase GtaA,** |  |
