# Supplementary material for: Mitochondrial Complex I Is a Global Regulator of Secondary Metabolism, Virulence and Azole Sensitivity in Fungi
Source: PLoS One. 2016 Jul 20;11(7):e0158724. doi: 10.1371/journal.pone.0158724 (PMC4954691; doi:10.1371/journal.pone.0158724)
Supplement: S5 Table — (DOCX) [file pone.0158724.s008.docx]

**S5 Table. Primers used in this study.**

| Primer ID | Sequence | Purpose |
| --- | --- | --- |
| 2g10600P1 | CCATTTGACATCCAGCCTCT | Generation of fusion KO cassette for gene 2G10600 |
| 2g10600P2 | TAGTTCTGTTACCGAGCCGGCCTGCGGAGTGATTTGTACG |  |
| 2g10600P3 | GCTCTGAACGATATGCTCCCGGCACCCATTCGAGACAATT |  |
| 2g10600P4 | CTGTACGTCTTCATCAGCGC |  |
| 2g10600P5 | GAGTATCAGACGGCTCGGAA |  |
| 2g10600P6 | CAGGTCGCTATCATACCGCT |  |
| 2g10600P7 | TTCCCAGAGTCCTCCGTCTA | Validation of Gene KO |
| 2g10600P8 | CTTCAATCAGACCAGCACCA |  |
| 2g10600KIP1 | CCATTTGACATCCAGCCTCT | Generation of reconstitution cassette for 2G10600 |
| 2g10600KIP2 | TAGTTCTGTTACCGAGCCGGCATGCGGCGCAACTATAAC |  |
| 2g10600KIP3 | GCTCTGAACGATATGCTCCCCCTGTGGAACGAGCCTGTAT |  |
| 2g10600KIP4 | TACGTCTTCATCAGCGCATT |  |
| 2g10600KIP5 | GTATCAGACGGCTCGGAAGA |  |
| 2g10600KIP6 | GCAATTGCAATCATTCAGGT |  |
| Linker_F | CCGGCTCGGTAACAGAACTAACGGCGTAACCAAAAGTCAC | Amplification of hph and zeo resistance markers for KO and reconstitution cassettes |
| Linker_R | GGGAGCATATCGTTCAGAGCTCTTGACGACCGTTGATCTG |  |
