# Supplementary material for: Mitochondrial Complex I Is a Global Regulator of Secondary Metabolism, Virulence and Azole Sensitivity in Fungi
Source: PLoS One. 2016 Jul 20;11(7):e0158724. doi: 10.1371/journal.pone.0158724 (PMC4954691; doi:10.1371/journal.pone.0158724)
Supplement: S6 Table — (DOCX) [file pone.0158724.s009.docx]

**S6 Table.** ***N. crassa* mutants used in this study**

| Knockout reference | *N. crassa* gene ID | Gene function |
| --- | --- | --- |
| FGSC12635, FGSC12636 | NCU09299.2 | B16.6 |
| FGSC13054, FGSC13055 | NCU01142.2 | B17.2 |
| FGSC12438, FGSC12439 | NCU04044.2 | Nuo 51 flavoprotein 1 |
| FGSC12432 | NCU02754.2 | Nuo49 |
| FGSC11169, FGSC11170 | NCU02814.2 |  |
| FGSC13361 | NCU01859.2 | 21 |
| FGSC13146, FGSC13147 | NCU01467.2 | NUO-14 NADHdehydrogenase (ubiquinone) |
| FGSC14867 | NCU03156.2 | NUO-10.5 |
| FGSC16064, FGSC16065 | NCU02280.2 | 21.3KD subunit |
| FGSC15972 | NCU00484.2 | 13K-A chain precursor |
| FGSC12564, FGSC12565 | NCU00160.2 | nuo6.6 |
| FGSC16348 | NCU01765.2 | 78K chain precursor |
| FGSC16392, FGSC16393 | NCU00670.2 | nuo9.5 |
| -no KO | NCU05299.2 | 29.9KD subunit, nuo29.9 |
